# Rapid and reversible fluorescent probe enables repeated snapshot imaging of AMPA receptors during synaptic plasticity

**DOI:** 10.1126/sciadv.adt6683

**Published:** 2025-06-06

**Authors:** Kyohei Soga, Takaaki Fujiwara, Mayu Nakagawa, Akihiro Shibata, Hansel Adriel, Kenji Yatsuzuka, Wataru Kakegawa, Michisuke Yuzaki, Itaru Hamachi, Eriko Nango, Shigeki Kiyonaka

**Affiliations:** ^1^ Department of Biomolecular Engineering, Graduate School of Engineering, Nagoya University, Nagoya, Aichi 464-8603, Japan.; ^2^ Institute of Multidisciplinary Research for Advanced Materials, Tohoku University, Sendai, Miyagi 980-8577, Japan.; ^3^ Department of Chemistry, Graduate School of Science, Tohoku University, Sendai, Miyagi 980-8578, Japan.; ^4^ Department of Physiology, Keio University School of Medicine, Tokyo 160-8582, Japan.; ^5^ Department of Synthetic Chemistry and Biological Chemistry, Graduate School of Engineering, Kyoto University, Kyoto 615-8510, Japan.; ^6^ RIKEN SPring-8 Center, Sayo, Hyogo 679-5198, Japan.; ^7^ Research Institute for Quantum and Chemical Innovation, Institutes of Innovation for Future Society, Nagoya University, Nagoya, Aichi 464-8603, Japan.

## Abstract

The subcellular localization of neurotransmitter receptors is strictly regulated in neurons. Changes in the trafficking of α-amino-3-hydroxy-5-methyl-4-isoxazolepropionic acid (AMPA)–type glutamate receptors (AMPARs) play an essential role in synaptic plasticity, which is the cellular basis of learning and memory. To explore receptor trafficking, genetically encoded approaches (e.g., the fusion of fluorescent proteins to receptors) are often used. However, concerns remain that genetic approaches cannot fully reproduce the receptor functions that are inherent to neurons. Herein, we report on PFQX1(AF488), a fluorescent probe for the visualization of cell-surface AMPARs without any genetic manipulation to neurons. The rapid and reversible staining features of this probe enabled snapshot imaging, which showed the accumulation of native AMPARs in dendritic spines during synaptic plasticity. Moreover, the mechanism of this synaptic accumulation, for which genetically encoded approaches have given controversial results, was revealed by integrating two chemical methods: PFQX1(AF488) and covalent chemical labeling.

## Teaser

A fluorescent probe, PFQX1(AF488), enables snapshot imaging of AMPA receptors during synaptic plasticity.

## INTRODUCTION

Synapses are the fundamental units of brain function; their structure and function are strengthened or weakened over time in response to a variety of inputs. This phenomenon is known as synaptic plasticity, which is a fundamental process that underlies learning and memory acquisition (*1*–*3*). In particular, excitatory postsynaptic currents mediated by ionotropic glutamate receptors are enhanced by repeated synaptic activity; this is known as long-term potentiation (LTP).

α-Amino-3-hydroxy-5-methyl-4-isoxazolepropionic acid (AMPA)–type glutamate receptors (AMPARs) are the most abundant ionotropic glutamate receptors. They play essential roles in learning and memory (*4*, *5*). AMPARs are localized not only on the postsynaptic terminal but also intracellularly and in extrasynaptic regions such as the dendritic surface. The subcellular localization of AMPARs is dynamically regulated by endocytosis, exocytosis, and lateral diffusion (*4*–*6*). Many studies have suggested that changes in AMPAR trafficking have essential roles in the expression of LTP (*7*–*9*). However, further clarification of these mechanisms is necessary to better understand the molecular mechanisms of memory.

To analyze AMPAR trafficking, fluorescent proteins can be fused to the extracellular domain of AMPARs using genetic approaches. Notably, a pH-sensitive variant of green fluorescent protein [super-ecliptic pHluorin (SEP)] has been widely used for visualizing cell-surface AMPARs in neurons (*7*, *8*, *10*, *11*). Self-labeling protein tags such as SNAP-tag (*12*) or Halo-tag (*13*, *14*) with cell-impermeable fluorescent probes have also been used instead of fluorescent proteins. Although these protein tags are undoubtedly powerful for studying AMPAR trafficking in neurons, they largely rely on the overexpression of genetically encoded receptors. Given that the number of AMPARs is strictly controlled at synapses (*4*, *15*), their expression levels, posttranslational modifications, and interactions with accessory proteins are likely to be affected by the overexpression of tagged AMPARs. To resolve this critical problem, a knock-in strategy has been reported (*16*–*18*) in which a protein tag is inserted into the N-terminal region of the target AMPAR subunit gene in the genome. However, the introduction of an exogenous gene into the genome may, in some cases, affect the transcription and/or stability of AMPAR mRNA (*16*). Furthermore, considering that the N-terminal region of ionotropic glutamate receptors interacts with synaptic proteins, the introduction of a large protein tag to this region may affect AMPAR function.

Ideally, native AMPARs would be analyzed without any genetic manipulation to clarify their physiological roles. In this context, we have previously reported the fluorescent labeling of cell-surface AMPARs endogenously expressed in neurons using ligand-directed two-step labeling (fig. S1) (*19*–*22*). In this method, in the first step, a trans-cyclooctene (TCO)–bearing chemical reagent, termed CAM2(TCO), is recognized by AMPARs, and amino acid residues near the ligand-binding domain (LBD) of AMPAR are covalently labeled with TCO (*22*). In the second step, TCO-labeled AMPARs on the cell surface were covalently labeled with fluorescent probes within 5 min using a fast click chemistry reaction. Although this method is powerful for tracking and analyzing cell-surface AMPARs, analyzing the contribution of intracellular AMPARs remains challenging. Given that AMPARs are dynamically regulated during synaptic plasticity through endocytosis and exocytosis, other methods capable of analyzing these processes are required.

In the present study, we report a fluorophore-ligand conjugate, termed PFQX1(AF488), for the rapid and reversible visualization of AMPARs in neurons. This probe enabled us to stain cell-surface AMPARs immediately after its addition to the medium. Owing to its reversible and reproducible staining features, PFQX1(AF488) was successfully used to visualize the trafficking of AMPARs, including the exocytosis step, during LTP. Furthermore, we were able to elucidate the mechanisms of this trafficking by combining PFQX1(AF488) with ligand-directed two-step labeling.

## RESULTS

### Development of fluorophore-ligand conjugates for visualizing cell-surface AMPARs

**Fig. 1.**
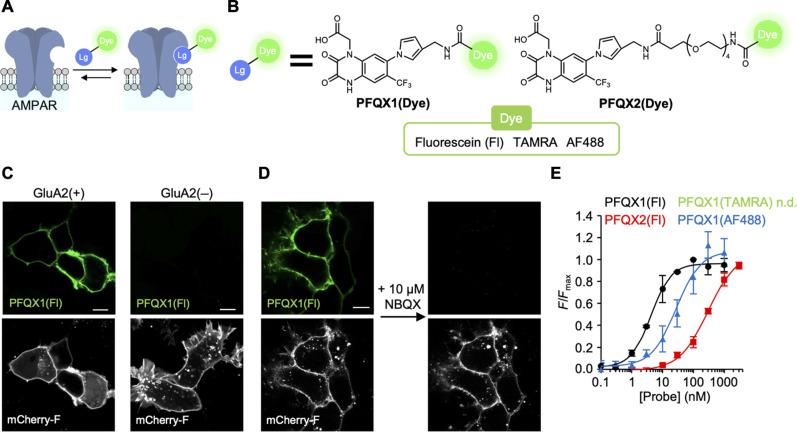
Development of fluorophore-ligand conjugates for AMPARs. (**A**) Schematic illustration of AMPAR staining with fluorophore-ligand conjugates. Lg, AMPAR ligand; Dye, fluorescent dye. (**B**) Chemical structures of fluorophore-ligand conjugates. See fig. S2 for detailed chemical structures. (**C**) Confocal live images of HEK293T cells transfected with GluA2 [GluA2(+)] or its control vector [GluA2(−)]. The cells were treated with 100 nM PFQX1(Fl). mCherry-F was used as a transfection marker. Scale bars, 10 μm. (**D**) Competitive inhibition of PFQX1(Fl) binding by NBQX. PFQX1(Fl) (100 nM) was added to the HEK293T cells expressed with GluA2. Then, 10 μM NBQX was added to the medium. Scale bar, 10 μm. (**E**) Concentration-dependent binding of fluorophore-ligand conjugates to GluA2 (*n* = 3). See fig. S3A for detailed results. Data are represented as means ± SEM. n.d., not determined.

To visualize AMPARs on the cell surface, we designed cell-impermeable fluorescent probes that were capable of binding to AMPARs (Fig. 1, A and B). For selective binding to AMPARs, we focused on 6-pyrrolyl-7-trifluoromethyl-quinoxaline-2,3-dione (PFQX), an anionic ligand with a high affinity [inhibition constant (*K*_i_) = 170 nM] that has been developed as a selective antagonist for AMPARs among glutamate receptors (*23*). Fluorescein (Fl), which has anionic, hydrophilic, and structurally compact features, was selected as the fluorophore. Next, Fl was fused to the pyrrole moiety of PFQX, according to the structural information of AMPARs with ZK200775 (*24*), a PFQX analog. We synthesized both PFQX1(Fl), in which PFQX and Fl were directly connected, and PFQX2(Fl), which contained an ethylene glycol linker between PFQX and Fl (Fig. 1B and fig. S2).

AMPARs form tetrameric cation channels that are composed of GluA1–GluA4 subunits (*25*), of which GluA2 is the most abundant (*26*). Using confocal microscopy, we then analyzed the binding of the synthesized probes to AMPARs in human embryonic kidney (HEK) 293T cells that transiently expressed GluA2. After the addition of 100 nM PFQX1(Fl) to the culture dish, prominent fluorescent signals were observed from the cell surface of GluA2-transfected cells but not from control cells (Fig. 1C). The fluorescent signals disappeared with the addition of 10 μM 2,3-dioxo-6-nitro-1,2,3,4-tetrahydrobenzo(f)quinoxaline-7-sulfonamide (NBQX), a competitive ligand (Fig. 1D), suggesting that the fluorescent signals were driven by ligand recognition. As shown in Fig. 1E, the fluorescent signals occurred in a probe concentration-dependent manner. Using PFQX1(Fl), cell-surface fluorescence was clearly observed at a probe concentration of 3 nM, and the signals were saturated at a 30 nM probe (Fig. 1E and fig. S3A). However, the addition of 30 nM PFQX2(Fl), which has an ethylene glycol linker, only led to a weak fluorescent signal from the cell surface, whereas prominent signals were obtained at concentrations of 100 nM and greater. Although the fluorescent signals of PFQX2(Fl) from the cell surface were saturated at 1000 nM, fluorescence became visible even in the medium. The binding affinity of the fluorophore-ligand conjugates with GluA2 was then determined from the cell-surface signal; the dissociation constant (*K*_d_) values for PFQX1(Fl) and PFQX2(Fl) were 4.3 ± 0.5 and 346 ± 99 nM, respectively (Fig. 1E). The affinity of PFQX1(Fl) was higher than those reported for both PFQX (*K*_i_ = 170 nM) (*23*) and PFQX-amine (*K*_i_ = 477 ± 171 nM), the synthetic intermediate of PFQX1(Fl) (fig. S3, B to D). This finding indicates that the fluorophore moiety of PFQX1(Fl) may contribute to increasing its affinity to GluA2.

To further explore the effects of the fluorophore on PFQX1(Dye) affinity, we synthesized PFQX1(TAMRA) and PFQX1(AF488) (Fig. 1B and fig. S2). The fluorescent visualization of AMPARs using these probes was evaluated in HEK293T cells, as described in the previous paragraph. With PFQX1(TAMRA), which has a cationic rhodamine fluorophore, only weak cell-surface fluorescence was visualized, even at a probe concentration of 100 nM (fig. S3A). Although the cell-surface fluorescent signal corresponding to AMPARs became more visible at 1000 nM, the fluorescence intensity from the extracellular solution was the same level as that from the cell surface. Conversely, with PFQX1(AF488), which has a rhodamine backbone and two sulfonate groups, cell-surface fluorescence was observed upon the addition of a relatively low probe concentration (10 nM). The *K*_d_ value of PFQX1(AF488) to GluA2 in HEK293T cells was 37.2 ± 9.8 nM, implying that the anionic xanthene dye in PFQX1(Dye) contributed to increasing the affinity to AMPARs (Fig. 1E and fig. S3A). The characters of these probes are summarized in table S1.

Given that neuronal activity may change pH homeostasis (*27*), we next tested the pH dependency of the fluorescence intensity of the two high-affinity probes [PFQX1(Fl) and PFQX1(AF488)] to AMPARs. As shown in fig. S4, the fluorescence intensity of PFQX1(Fl) was weakened even under mild acidic conditions (e.g., pH 6), whereas that of PFQX1(AF488) was relatively unaffected at pH 5 to 10. Therefore, PFQX1(AF488) likely enables the quantification of cell-surface AMPARs without being affected by pH changes in extracellular spaces.

### Structural analysis of the fluorophore-ligand conjugate with AMPARs

**Fig. 2.**
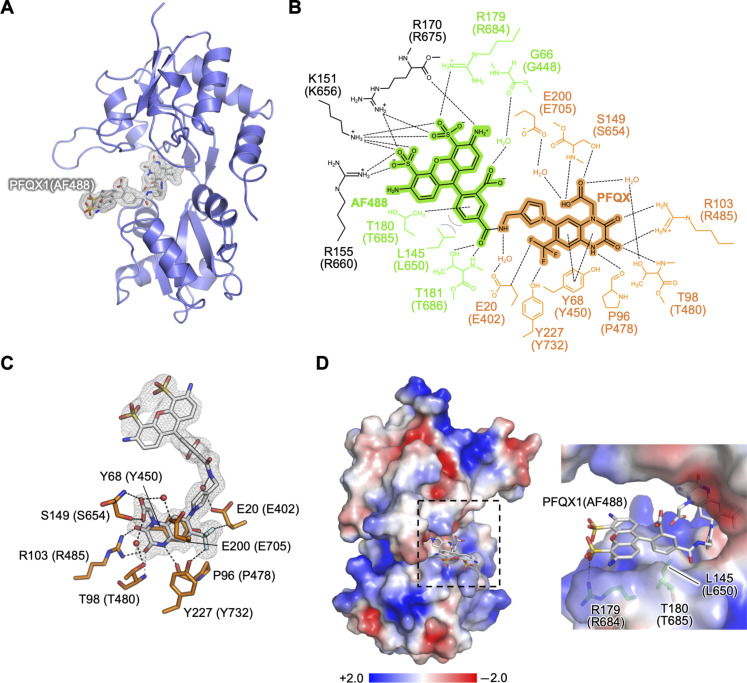
Structure of the S1S2J complexed with PFQX1(AF488). The residue numbers of S1S2J are shown, and the corresponding residue of full-length GluA2 is also shown in parentheses. (**A**) The overall structure of the S1S2J bound to PFQX1(AF488) is displayed as a ribbon diagram. The electron density of bound PFQX1(AF488) (gray) was shown as the polder map at a contoured level of 3.0σ. (**B**) Interaction manner of the PFQX1(AF488). The PFQX and AF488 parts of the PFQX1(AF488) are highlighted in orange and green, respectively. The residues and waters contacted to the PFQX and AF488 are also shown in orange and green. The interacting residues in the symmetry mate are colored in black. Polar and hydrophobic interactions are shown as dotted lines (black) and solid lines (gray), respectively. (**C**) Contacts between the PFQX part and S1S2J. The residues interacted with the PFQX part are shown as sticks (orange). The polder map of the PFQX1(AF488) is shown at 3.0σ. (**D**) Adaptive Poisson-Boltzmann Solver (APBS)-generated electrostatic potential of the S1S2J. A positively charged surface around the AF488 is extended. Right: Close-up view of the ligand binding site, and the residues involved in recognizing the AF488 part are shown as sticks (green).

As described in the previous section, the affinity of PFQX1(AF488) to AMPARs was one order of magnitude higher than that of the original ligand PFQX. To elucidate the binding manner of PFQX1(AF488), we conducted structural analyses of AMPAR bound to PFQX1(AF488). AMPARs form tetrameric ion channels, and each subunit is composed of an N-terminal domain, an LBD, and a transmembrane domain (fig. S5A) (*25*). Competitive antagonists, including PFQX, bind to the LBD (*20*, *23*). Therefore, we used a ligand-binding core moiety of the GluA2 LBD, termed S1S2J (*28*, *29*), for the x-ray crystal structural analysis. The structure of S1S2J bound to PFQX1(AF488) was determined at 1.82 Å (Fig. 2A and table S2). In the structural representation, the residue numbers of S1S2J are shown and the corresponding residue of full-length GluA2 is shown in parentheses.

Many studies have reported that GluA2 LBD and S1S2J maintain an open form when the antagonist binds to the hinge region of its clamshell-like architecture (*29*, *30*). In the obtained S1S2J-PFQX1(AF488) structure, the S1S2J region showed an open form, which was almost identical to the previously reported structure bound with NBQX (a well-known antagonist) (*31*), with a root mean square deviation of 0.64 Å for 258 C^α^ atoms (fig. S5B). In the structure, PFQX1(AF488) was recognized over a large area of the aperture of S1S2J (Fig. 2B and table S3).

The PFQX part of PFQX1(AF488) was deeply bound to the aperture of S1S2J and showed a similar binding mode to that of ZK200775 (*32*), which is a structural analog of PFQX (Fig. 2C and fig. S5C). Specifically, the aromatic group of PFQX was stacked with Tyr^68^ (Tyr^450^ in full-length GluA2). The two carbonyl groups of PFQX, which mimic the α-carboxyl group of glutamate, formed hydrogen bonds with Arg^103^ (Arg^485^) and Thr^98^ (Thr^480^). In contrast to the PFQX part, the AF488 fluorophore part of PFQX1(AF488) was located at the vestibule of the binding cleft of S1S2J. In addition, the phenyl group of AF488 was situated at the hydrophobic region surrounded by Leu^145^ (Leu^650^) and Thr^180^ (Thr^685^). Thr^180^ (Thr^685^) formed a CH-π interaction with the phenyl group. The main-chain amide group and the side chain of Thr^181^ (Thr^686^) formed a hydrogen bond with the carbonyl group of the amide group of the AF488 part (Fig. 2B). Of note, the negatively charged sulfonate group of AF488 interacted with the side chain of Arg^179^ (Arg^684^) (Fig. 2D). Furthermore, the side chain of Lys^67^ (Lys^449^) and the main-chain amide bonds consisting of Asp^146^ (Asp^651^)–Ser^147^ (Ser^652^)–Gly^148^ (Gly^653^) (referred to as the DSG loop) provided an electropositive environment that was favorable to locating the sulfonate group of AF488 (Fig. 2D and fig. S5D). Therefore, the x-ray structural analysis clearly revealed the molecular basis for the increased affinity of PFQX1(AF488) bearing an anionic fluorophore for AMPARs.

### Rapid and reversible staining of cell-surface AMPARs in HEK293T cells

**Fig. 3.**
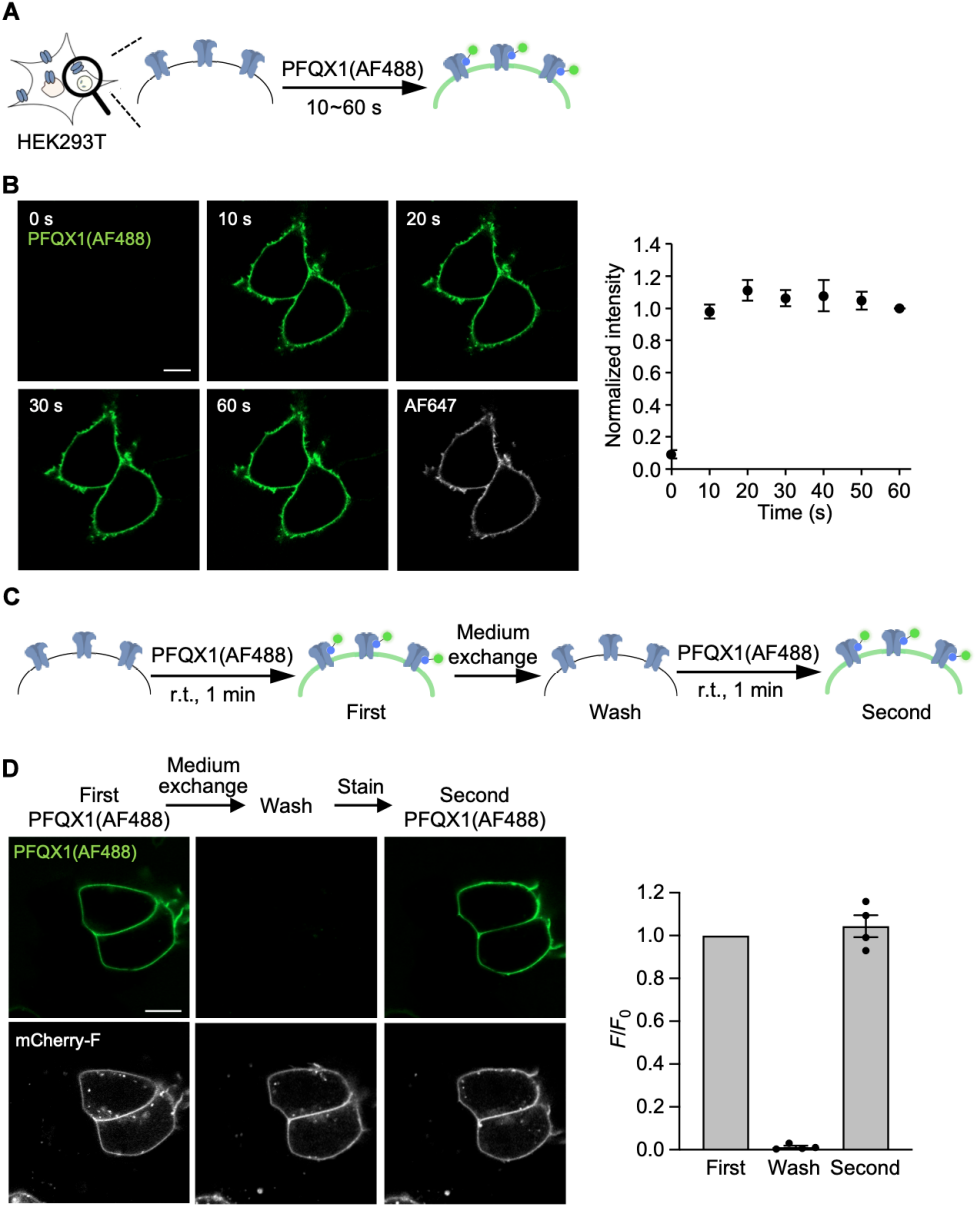
Rapid and reversible staining of AMPARs in HEK293T cells. (**A**) Experimental procedure to analyze the time course of PFQX1(AF488) binding to GluA2 on live cells. The fluorescent images were obtained at specified time points after the addition of 100 nM PFQX1(AF488) by confocal microscopy. (**B**) Left: Representative confocal live images of HEK293T cells expressing Halo-tag-fused GluA2 on the N terminus after the addition of 100 nM PFQX1(AF488). Halo-tag ligand AF647 was used as a transfection marker. Right: The surface intensity of AF488 was quantified, which was normalized to the intensity at 60 s (*n* = 3). Scale bar, 10 μm. (**C**) Experimental procedure to analyze the reversibility of PFQX1(AF488) binding to GluA2. First image was obtained 1 min after addition of 100 nM PFQX1(AF488) to the culture medium. A wash image was obtained after the medium exchange. Then, 100 nM PFQX1(AF488) was re-treated to obtain the second image. r.t., room temperature. (**D**) Left: Representative confocal live cell imaging of HEK293T cells expressed with GluA2 to analyze the reversibility of PFQX1(AF488) binding. mCherry-F was used as a transfection marker. Right: The surface intensity of AF488 was quantified, which was normalized to the first images (*n* = 4). Scale bar, 10 μm. Data are represented as means ± SEM.

After developing PFQX1(AF488), a high-affinity probe for AMPARs, we applied this probe for the fluorescent imaging of AMPAR in living cells. AMPARs are expressed not only on the cell surface but also intracellularly. However, confocal imaging revealed that PFQX1(AF488) selectively bound to cell-surface AMPARs (fig. S6). To quantitatively analyze the amount of cell-surface AMPARs in each period during live imaging, this fluorescent staining had to occur in a rapid, reversible, and reproducible manner. We analyzed the kinetics of PFQX1(AF488) binding to AMPARs in HEK293T cells expressing GluA2 (Fig. 3A). As shown in Fig. 3B, prominent fluorescence was observed from the cell surface just 10 s after the addition of 100 nM PFQX1(AF488) to the culture dish. This fluorescence intensity was saturated within 20 s after the addition of the probe, demonstrating its rapid binding kinetics.

To evaluate reversibility, after AMPARs were visualized using 100 nM PFQX1(AF488), the washing out of bound PFQX1(AF488) was examined via medium exchange (Fig. 3C). Subsequently, the same concentration of probe was added again to the dish to confirm reproducibility. As shown in Fig. 3D, fluorescence from the cell surface was lost after the medium exchange in the same region of view, demonstrating the reversibility of PFQX1(AF488) binding to AMPARs. When 100 nM PFQX1(AF488) was added again to the dish, prominent fluorescence from the cell surface was observed at the same level as that obtained after the first treatment. Together, these results indicate that cell-surface AMPAR levels can be quantified repeatedly, even after replacing the culture medium.

AMPARs form tetrameric ion channels composed of GluA1–4 subunits (*25*), and PFQX antagonized AMPARs in a subunit-nonspecific manner (fig. S7A). Consistently, the addition of PFQX1(AF488) led to the visualization of all AMPAR subunits (GluA1–4) (fig. S7, B and C, and table S1). PFQX binds to AMPARs with high affinity but is also known to weakly bind to kainate receptors, a type of ionotropic glutamate receptor (*23*). We then examined the effect of PFQX1(AF488) on GluK2, an abundant kainate receptor subunit in the hippocampus. As shown in fig. S8, the inhibitory effect of PFQX-amine on GluK2 is low, and PFQX1(AF488) failed to visualize GluK2, even at its high concentration (300 nM). These findings indicate that the probe can be used to visualize AMPAR regardless of subunit type.

### Visualization of endogenous AMPARs in living hippocampal neurons

**Fig. 4.**
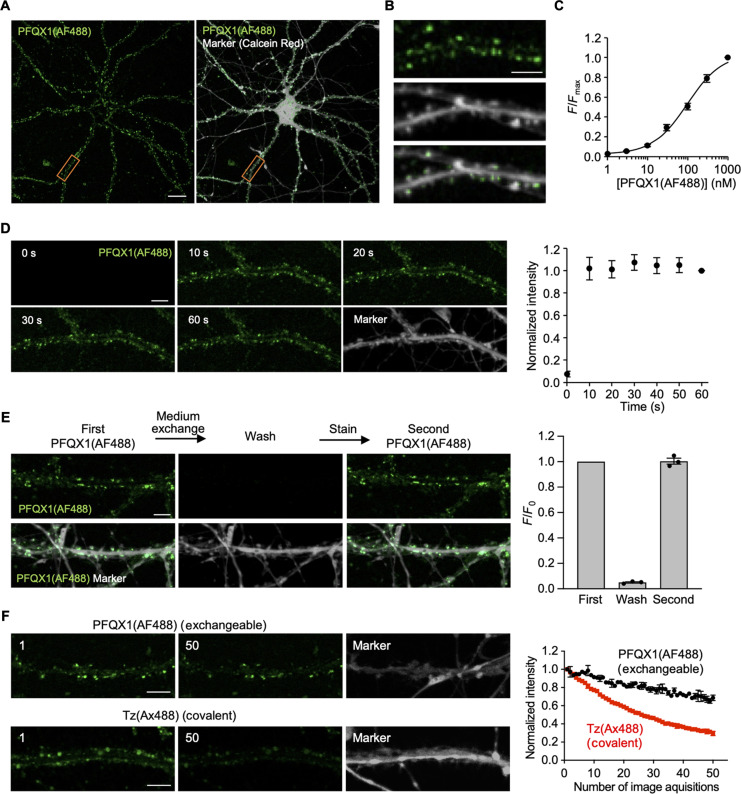
Visualization of native AMPARs in cultured hippocampal neurons. (**A** and **B**) Confocal live images of cultured hippocampal neurons treated with 100 nM PFQX1(AF488). Calcein red signals are shown in gray as a cytoplasmic marker. Orange ROIs indicated in (A) are expanded in (B). Scale bars, 20 μm in (A) and 5 μm in (B). (**C**) Concentration dependency of PFQX1(AF488) binding to AMPARs in cultured hippocampal neurons (*n* = 3). The *K*_d_ value was 97.5 ± 9.9 nM. (**D**) Time course of PFQX1(AF488) binding. The cells were treated with 100 nM PFQX1(AF488), and images were obtained at specified time points by confocal microscopy. Left: Representative confocal live images of cultured hippocampal neurons after the addition of 100 nM PFQX1(AF488). Right: The intensity of PFQX1(AF488) in spines was quantified, which was normalized to the intensity at 60 s (*n* = 3). Scale bar, 5 μm. (**E**) Reversibility of PFQX1(AF488) binding to AMPARs. Left: Representative confocal live imaging of cultured hippocampal neurons. Right, the intensity of PFQX1(AF488) in spines was quantified, which was normalized to the first images. [PFQX1(AF488)] = 100 nM (*n* = 3). Scale bar, 5 μm. (**F**) Repeated confocal imaging of cultured hippocampal neurons. Left: Representative images. The number of repeated acquisitions is indicated in the images. Right, the intensity of AF488 in spines was analyzed and normalized to the intensity in the first image (*n* = 3). [PFQX1(AF488)] = 30 nM. Scale bars, 5 μm. Data are represented as means ± SEM.

We next evaluated PFQX1(AF488) for the visualization of endogenous AMPARs in primary cultured hippocampal neurons. To this end, a cytosolic marker that is applicable to live cell imaging (CellTracker Red or Calcein Red-AM) was added to the culture medium to stain the intracellular region of the neurons. After the addition of 100 nM PFQX1(AF488), fluorescent puncta of PFQX1(AF488) were observed along dendrites in the hippocampal neurons pretreated with the cytosolic marker (Fig. 4, A and B). Immunostaining for postsynaptic density 95 (PSD95) gave similar punctate signals along dendrites (fig. S9A), suggesting that the PFQX1(AF488) puncta corresponded to dendritic spines in the neurons. Furthermore, the fluorescent signals from the puncta disappeared with the addition of 10 μM NBQX, a competitive ligand (fig. S9B), further indicating that the fluorescent puncta corresponded to the AMPAR signals.

We next conducted a more detailed investigation into the binding properties of PFQX1(AF488) to AMPARs in cultured hippocampal neurons. As shown in fig. S9C, fluorescent signals of PFQX1(AF488) were observed in a concentration-dependent manner. The *K*_d_ value of PFQX1(AF488) in neurons was 97.5 ± 9.9 nM (Fig. 4C), which was slightly higher than that obtained in HEK293T cells transfected with AMPARs (37.2 ± 9.8 nM) (fig. S7C and table S1). In neurons, the auxiliary subunits play key roles in regulating the trafficking, scaffolding, and channel properties of AMPARs (*33*, *34*). Recent structural analyses have revealed that the extracellular loop of the auxiliary subunits, such as GSG1L and type II TAPRs, is located near the LBD of GluA2 (*35*, *36*). We then examined the effects of the auxiliary subunits (GSG1L and TARP γ7, a type II TARP subtype) on PFQX1(AF488) binding in HEK293T cells. The *K*_d_ value of PFQX1(AF488) was slightly shifted toward higher concentration in the presence of TARP γ7 but not GSG1L (fig. S10). The *K*_d_ value (118.3 ± 44.7 nM) in the presence of TARP γ7 in HEK293T cells was comparable to that (97.5 ± 9.9 nM) obtained in cultured neurons (table S1). These results indicate that the difference in the affinity between HEK293T cells and neurons may be due to the presence of auxiliary subunits of AMPARs in neurons.

As was the case in HEK293T cells, the binding kinetics of PFQX1(AF488) to AMPARs were rapid, and the fluorescent signals were saturated after 10 s of probe addition to the medium (Fig. 4D). As shown in Fig. 4E, the fluorescence was easily removed by medium exchange, and re-treatment with PFQX1(AF488) produced fluorescence intensity at the same level as that of the first treatment. Conceptually, this reversible binding feature allows for the exchange of the probe even after its fluorophore has been photobleached. As shown in Fig. 4F, in the case of covalent labeling with the AF488 dye (shown as Ax488) using ligand-directed two-step labeling, repeated acquisition of the fluorescent images resulted in a decrease in fluorescence. In contrast, for PFQX1(AF488), fluorescent decreases were markedly suppressed under the same conditions. These results indicate that PFQX1(AF488) can be used to visualize endogenous AMPARs in cultured hippocampal neurons in a rapid, reversible, and reproducible manner.

We also examined the applicability of this probe for staining endogenous AMPARs in acutely prepared brain slices. Here, we focus on the cerebellum, because AMPARs are highly expressed in the molecular layer, which allows confirmation of AMPAR-binding in the slices (fig. S11A). After a 5-min incubation of PFQX1(AF488) with acutely prepared cerebellar slices in artificial cerebrospinal fluid (ACSF), strong fluorescence was observed in the molecular layer of the cerebellar slices (fig. S11B). This fluorescence is significantly weakened in the copresence of NBQX, a competitive inhibitor, suggesting that the fluorescent signal corresponds to AMPARs (fig. S11, C and D). We also examined the reversibility of probe binding by medium exchange in the slices. Although the incubation time is required for at least 5 min, PFQX1(AF488) can be washed out by the medium exchange (fig. S11, E and F). Thus, this probe can be applicable to visualize AMPARs not only in cultured neurons but also in acutely prepared brain slices.

### Fluorescent visualization of cell-surface AMPARs during synaptic plasticity

Many studies have reported that neuronal AMPAR trafficking is strictly regulated during synaptic activity (*7*–*9*). In particular, AMPAR accumulation at the postsynaptic terminal has been observed with LTP stimulation. However, the current techniques largely depend on the fusion of fluorescent proteins to AMPARs, and in most cases, the fusion proteins are overexpressed in neurons. Ideally, endogenous AMPARs should be visualized under live conditions with minimal perturbation. Taking advantage of the unique binding features of PFQX1(AF488), we used PFQX1(AF488) to investigate endogenous AMPAR trafficking in neuronal cells before and after LTP stimulation.

**Fig. 5.**
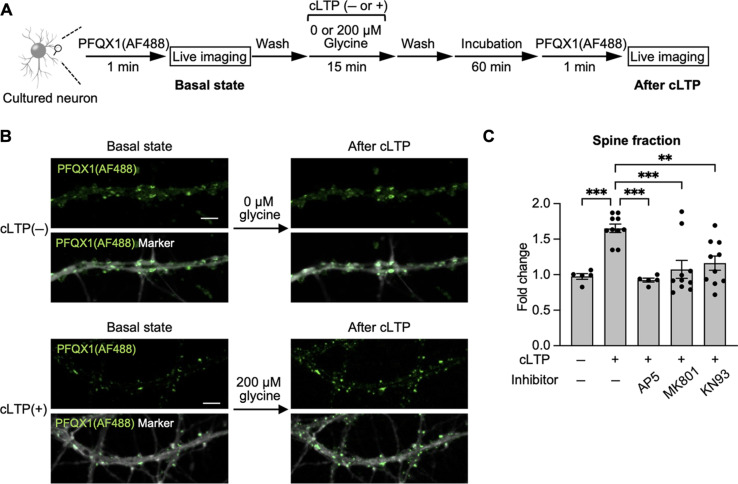
Visualization of the surface accumulation of AMPARs by cLTP stimulation. (**A**) Experimental scheme of cLTP stimulation. “Basal state” images were obtained after the addition of 100 nM PFQX1(AF488) to cultured hippocampal neurons. The probe was washed out by medium exchange. Then, cLTP was induced in cLTP stimulation buffer containing 200 μM glycine at room temperature for 15 min. After medium exchange with cLTP incubation buffer, the cells were further incubated for 60 min. Then, the cells were treated again with 100 nM PFQX1(AF488) to obtain “after cLTP” images. (**B**) Representative images of the cLTP experiment. Top: Images of the control condition “cLTP(−)” where glycine was not added to the cells are shown. Bottom: Images of the cells stimulated with glycine, “cLTP(+)”, are shown. Calcein red signals are shown in gray as a cytoplasmic marker. Scale bars, 5 μm. (**C**) The intensity of PFQX1(AF488) in spines was analyzed by the intensity of “after cLTP” images divided by that of “basal state” images. [AP5] = 50 μM, [MK-801] = 20 μM, and [KN93] = 5 μM (*n* = 5 to 10). Significant difference (**P < 0.01; ***P < 0.001, one-way ANOVA with Dunnett’s test). Data are represented as means ± SEM.

In the hippocampus, LTP is initiated by the activation of *N*-methyl-d-aspartate–type glutamate receptors (NMDARs) in response to repetitive neuronal stimulation (*1*, *37*, *38*). Selective activation of NMDARs using chemical compounds can also induce LTP (*39*, *40*), which is known as chemically induced LTP (cLTP). Here, we used PFQX1(AF488) to investigate cell-surface AMPAR levels before and after cLTP stimulation. As shown in Fig. 5A, cell-surface AMPARs were stained with PFQX1(AF488), and fluorescent images were obtained as the basal state using confocal microscopy. Following the removal of PFQX1(AF488) by medium exchange, cLTP stimulation was applied. To induce cLTP, Mg^2+^ in the medium was withdrawn in conjunction with the addition of 200 μM glycine, a co-agonist of NMDAR, for 15 min for the selective activation of NMDAR. After medium exchange to the incubation buffer and 1 hour of incubation, PFQX1(AF488) was added again to stain cell-surface AMPARs; fluorescent images after cLTP stimulation were then obtained using confocal microscopy (Fig. 5B). The fluorescence intensity of PFQX1(AF488) in the spines was significantly enhanced after cLTP stimulation, with a 1.6-fold increase compared with the basal state (Fig. 5C). By contrast, in cells without cLTP stimulation, the fluorescence intensity of PFQX1(AF488) did not obviously change after 1 hour of incubation. A recent study has suggested that remodeling of the synaptic microenvironment occurs during LTP, which may affect the ligand-binding properties of AMPARs or alter the pH of synaptic regions (*27*). In this context, the affinity of PFQX1(AF488) to AMPARs was unchanged even after cLTP stimulation (fig. S12A). In addition, the fluorescence intensity of PFQX1(AF488) was nearly unaffected at pH 6 to 8 in both HEK293T cells and cultured neurons (fig. S13), which is consistent with the results obtained in test tubes (fig. S4). These data suggest that the enhancement of fluorescence intensity after cLTP stimulation may be attributed to an increase in the number of AMPARs at the synaptic terminal.

Both NMDAR activation and the subsequent phosphorylation of synaptic proteins by calcium- and calmodulin-dependent protein kinase II (CaMKII) are critical steps in the expression of LTP (*1*, *37*, *38*, *41*, *42*). We therefore examined whether the increase in PFQX1(AF488) fluorescence after cLTP stimulation correlated with the activation of both NMDARs and CaMKII. As shown in Fig. 5C, pretreatment with NMDAR blockers (AP5 or MK-801) or a CaMKII inhibitor (KN93) suppressed the increase in PFQX1(AF488) fluorescence, even after cLTP stimulation. This result indicates that the observed increase in PFQX1(AF488) fluorescence strongly correlated with Ca^2+^ influx through NMDARs and the resulting downstream pathway.

One possible mechanism for the observed increase in the number of AMPARs on synaptic membranes is an elevation in AMPAR expression. We therefore quantified AMPAR expression levels before and after cLTP stimulation using Western blotting. As shown in fig. S12B, there were no significant changes in the expression levels of GluA1 or GluA2 (the main subunits of AMPARs in the hippocampus), even after cLTP stimulation (*43*). Therefore, the increased fluorescence intensity of PFQX1(AF488) in the spines could likely be attributed to altered AMPAR trafficking in neurons.

### Quantitative analysis of AMPAR trafficking during synaptic plasticity

**Fig. 6.**
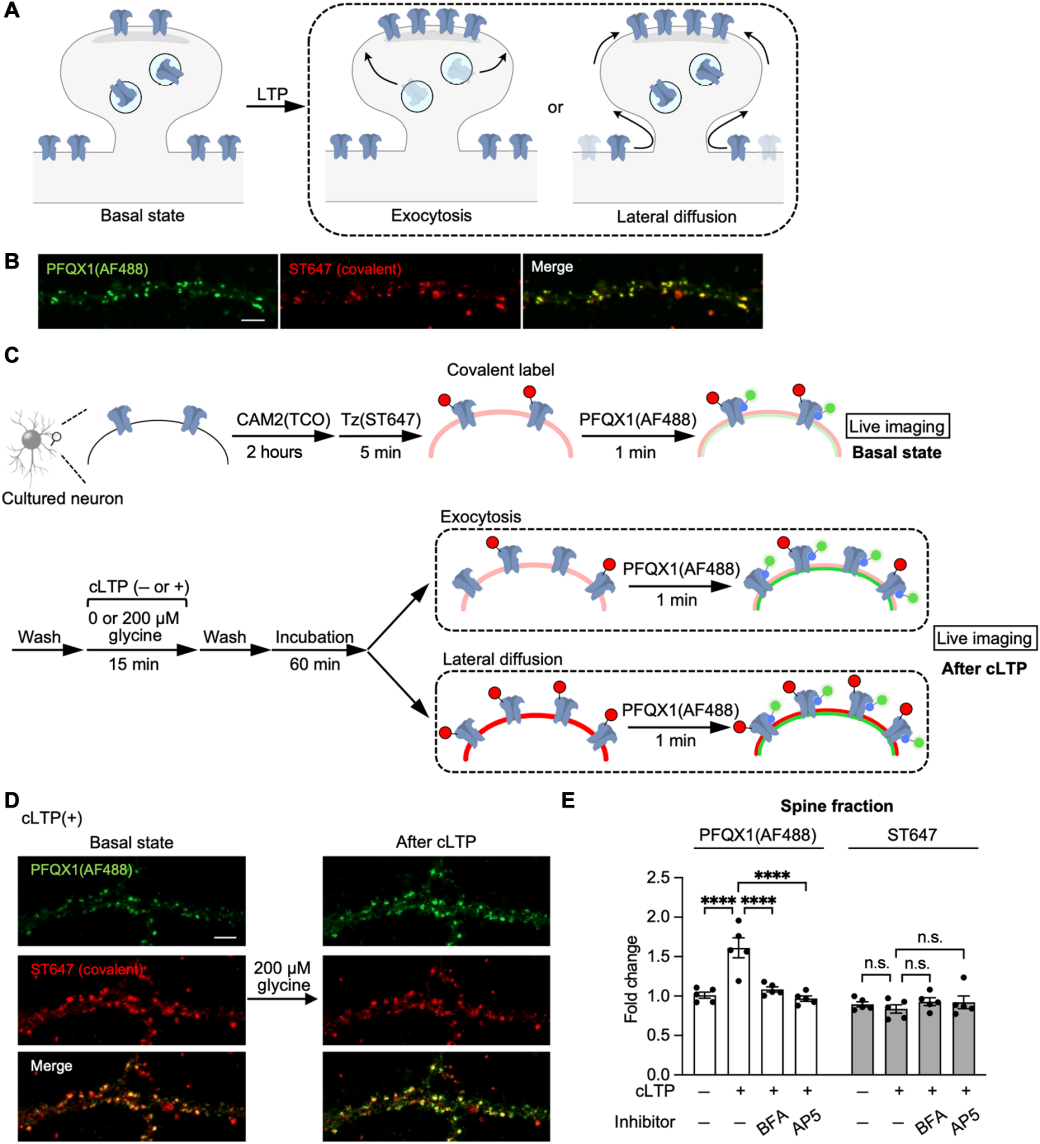
Mechanisms of the surface accumulation of AMPARs by cLTP stimulation. (**A**) Putative mechanisms of AMPAR accumulation in synapses during LTP. (**B**) Confocal live imaging of cultured hippocampal neurons. The cells were treated with 2 μM CAM2(TCO) for 2 hours, followed by the addition of 100 nM Tz(ST647) for 5 min. Then, 100 nM PFQX1(AF488) was added to the cells. A detailed scheme of two-step labeling of ST647 is shown in fig. S14A. Scale bar, 5 μm. (**C**) A schematic illustration of the experimental procedure for discriminating between exocytosis or lateral diffusion is shown. Cultured hippocampal neurons were covalently labeled with 2 μM CAM2(TCO) for 2 hours followed by 100 nM Tz(ST647) for 5 min. “Basal state” images were scanned after the addition of 100 nM PFQX1(AF488). After washing out of PFQX1(AF488) by medium exchange, cLTP was induced by 200 μM glycine for 15 min, and the cells were further incubated for 60 min in cLTP incubation buffer. The cells were treated with 100 nM PFQX1(AF488) again to obtain “after cLTP” images. A schematic illustration of putative results regarding ST647 labeling is also shown in fig. S14C. (**D**) Representative live cell images of the cLTP experiment. Images of the cells stimulated with glycine are shown. Scale bar, 5 μm. (**E**) The intensity of AF488 and ST647 in spines was analyzed. Fluorescence intensity of “after cLTP” images was divided by that of “basal state” images. Raw images for cLTP(−), brefeldin A (BFA) pretreatment, and AP5 pretreatment are shown in figs. S14D and S15 (B and C), respectively. [AP5] = 50 μM, [BFA] = 25 μM (*n* = 5). Significant difference (****P < 0.0001). n.s., not significant (*P* > 0.05, two-way ANOVA with Tukey’s test). Data are represented as means ± SEM.

For the delivery of AMPARs to synaptic membranes, two possible pathways have been proposed: the insertion of proteins from intracellular spaces by exocytosis (*44*–*46*) and the lateral diffusion of proteins from the surface of dendritic regions (*11*, *47*, *48*) (Fig. 6A). However, the contribution of both pathways during LTP remains controversial (see Discussion for details). In the present study, the use of PFQX1(AF488), which enabled the visualization of cell-surface AMPARs before and after cLTP stimulation, revealed increased AMPARs in the spines after cLTP stimulation (Fig. 5). Although this technique is powerful for visualizing cell-surface AMPARs in each period, it does not give any information about the trafficking pathway. We have previously reported a method for covalent labeling of cell-surface AMPARs, termed ligand-directed two-step labeling (fig. S1). Although this method cannot provide information regarding intracellular AMPARs, it is useful for tracking labeled AMPARs on plasma membranes. Taking advantage of the key features of each method, we combined PFQX1(AF488) with the two-step labeling method to elucidate the trafficking mechanism of AMPARs during cLTP.

Before analyzing AMPAR trafficking, we examined the feasibility of using PFQX1(AF488) in conjunction with the two-step labeling method. To demonstrate dual staining, cell-surface AMPARs were covalently labeled with the fluorophore, SeTau-647 (ST647), by two-step labeling; specifically, cultured hippocampal neurons were labeled with CAM2(TCO) followed by treatment with 100 nM Tz(ST647) for 5 min (fig. S14A). After the two-step labeling, the same neurons were stained with 100 nM PFQX1(AF488). As shown in Fig. 6B, the fluorescent signal of PFQX1(AF488) merged well with that of ST647. Furthermore, immunostaining of the neurons after fixation showed that the ST647 signals were observed alongside the dendrite marker microtubule-associated protein 2 (MAP2) (fig. S14B), which merged well with the postsynaptic marker PSD95. Together, these findings suggest that AMPARs were selectively labeled with ST647 and indicate that the two-step labeling method with PFQX1(AF488) staining can be used to simultaneously visualize AMPARs in living hippocampal neurons.

Next, we analyzed the changes in AMPAR trafficking after cLTP stimulation by combining the two-step labeling and PFQX1(AF488). The experimental procedure and two possible results are shown in Fig. 6C. If exocytosis is the main mechanism of synaptic accumulation, unlabeled AMPARs would increase at the synaptic surface, resulting in increased PFQX1(AF488) signals but no changes in ST647 intensity (Fig. 6C and fig. S14C). Conversely, if lateral diffusion is the main mechanism, labeled AMPARs would enter synapses from extrasynaptic regions, resulting in increases in both ST647 and PFQX1(AF488) fluorescence in the spines. As shown in Fig. 6 (D and E), cLTP stimulation increased the fluorescence of PFQX1(AF488), but it failed to increase ST647 fluorescence in the spines. In the absence of cLTP stimulation, neither PFQX1(AF488) nor ST647 showed noticeable changes in fluorescence intensity (Fig. 6E and fig. S14D). These results suggest that exocytosis is likely the primary pathway for AMPAR accumulation.

We further examined the trafficking mechanisms in detail. First, we analyzed the fluorescent signals from the entire cell surface, including not only dendritic spines but also dendritic shafts. As shown in fig. S15A, even in the total fluorescence, a prominent increase was observed for PFQX1(AF488) but not for the covalently labeled ST647 signals. This suggests that the number of AMPARs on the cell surface increases after cLTP stimulation. Next, to confirm the contribution of exocytosis to the fluorescent increase, we used brefeldin A, a potent inhibitor of protein transport, including exocytosis (*49*, *50*). As shown in fig. S16, brefeldin A markedly suppressed the spontaneous exocytosis of Halo-tag-fused AMPAR to the cell surface in HEK293T cells. In cultured hippocampal neurons, pretreatment with brefeldin A inhibited the fluorescent increase in PFQX1(AF488) even after cLTP stimulation (Fig. 6E and fig. S15B). These results support the idea that the fluorescent increase in PFQX1(AF488) originates from the exocytosis of intracellular AMPARs.

In our experiments, 100 nM PFQX1(AF488) is added prior to cLTP stimulation. Although the probe is washed away after acquisition of the image, we cannot exclude the possibility that acute inhibition of AMPARs by PFQX1(AF488) may affect the trafficking behavior of AMPARs. To minimize the inhibitory effects on AMPARs, the probe concentration was reduced to 10 nM, which would bind to less than 10% of AMPARs (Fig. 4C). As shown in fig. S17, even under this condition, a fluorescent increase in PFQX1(AF488) was observed after cLTP stimulation, with a similar level to that obtained using the 100 nM probe. Thus, acute inhibition of AMPARs over a short period is unlikely to affect the trafficking of AMPARs after cLTP.

Pretreatment with the NMDAR inhibitor AP5, an increase in both PFQX1(AF488) and ST647 fluorescence was not observed even after cLTP stimulation (Fig. 6E and fig. S15C). Moreover, as shown in fig. S18, no significant fluorescence changes were observed even when AMPARs were covalently labeled with AF488 using two-step labeling with Tz(Ax488) instead of Tz(ST647). This result indicated that the differences in fluorescence changes after cLTP stimulation between PFQX1(AF488) and ST647 labeling were unlikely to be caused by differences in fluorophores. Overall, our results strongly indicate that exocytosis is the main mechanism of AMPAR accumulation in dendritic spines during cLTP in cultured hippocampal neurons.

## DISCUSSION

In the present study, we developed PFQX1(AF488) for rapid and reversible staining of AMPARs, which enabled us to quantify cell-surface AMPARs in living cells. Using PFQX1(AF488), AMPAR accumulation in dendritic spines during synaptic plasticity was successfully visualized by obtaining images both before and after cLTP stimulation in cultured hippocampal neurons. Furthermore, the trafficking mechanism of AMPARs during synaptic plasticity was quantitatively evaluated using both PFQX1(AF488) and the covalent labeling of ST647 to cell-surface AMPARs. This analysis revealed that cLTP-induced AMPAR accumulation in dendritic spines was mainly mediated by exocytosis from the intracellular compartment rather than by lateral diffusion on the cell surface.

The affinity of PFQX1(Dye) to AMPARs was highly dependent on the structure of the conjugated fluorophore; both PFQX1(Fl) and PFQX1(AF488) had high affinity, whereas PFQX1(TAMRA) had low affinity. These differences were revealed in the current study via an x-ray crystallographic analysis of the LBD of AMPAR complexed with PFQX1(AF488). In the x-ray structure, AF488 formed hydrophobic and electrostatic interactions with the LBD. Consistent with the structural analysis results, the introduction of a linker between the fluorophore and the PFQX ligand in PFQX2(Fl) resulted in a marked decrease in AMPAR affinity. This structural information provides important insight for the molecular design of fluorescent probes for AMPARs, such as probes with long-wavelength-emitting dyes that are suitable for visualizing endogenous AMPARs in brain tissue or in vivo. Regarding the affinity of PFQX1(AF488) for each AMPAR subunit, its affinity for GluA1 is slightly lower than for GluA2–4. Our x-ray structural analysis of the GluA2 LBD suggested that the electropositive environment provided by the main-chain amide bonds in the DSG loop and Lys^67^ contributes to probe binding. However, the DSG loop is not conserved for GluA1 but is conserved in GluA2–4, which may account for the slight decrease in its affinity for GluA1.

The primary advantage of using PFQX1(AF488) is its simplicity in visualizing and quantifying cell-surface AMPARs without the need for any genetic manipulation. With this method, cell-surface AMPARs can be visualized immediately after the addition of the probe, not only in cultured neurons but also in brain slices. In addition, given that AMPARs are highly concentrated in dendritic spines, this probe can be used to directly visualize synapses in neurons. Another advantage of this probe is its reversible binding, which allows for capturing snapshot images of cell-surface AMPARs before and after synaptic activity changes. Regarding the limitations of PFQX1(AF488), because it is an antagonist-based probe, the function of stained AMPARs is inhibited during visualization, and glutamate-induced trafficking changes of AMPARs may be interfered. However, this antagonistic effect can be minimized by using a low concentration of this probe, such as 10 nM, under which less than 10% of AMPARs are suppressed. Even at this low concentration of the probe, AMPAR accumulation was successfully visualized, as shown in fig. S17. When applying this method to brain slices, some additional limitations arise. One issue is that the washing procedure takes time, requiring over 5 min to remove the probe. In addition, because of the high density of excitatory synapses, distinguishing individual synapses in brain tissue can be challenging. Despite these limitations, this method remains a powerful tool for quantifying the density of AMPARs in brain slices.

In the present study, PFQX1(AF488), bearing AF488 dye, was selected as a probe for visualizing AMPARs in hippocampal neurons because it enabled us to visualize the receptors in a pH-independent manner in the pH 5 to 10 range. Another probe, PFQX1(Fl), bearing Fl, also showed high affinity to AMPARs. However, in contrast to PFQX1(AF488), the fluorescence intensity of PFQX1(Fl) was weakened under acidic conditions (pH < 6). This property will be advantageous for evaluating AMPAR dynamics during long-term depression, which is another form of synaptic plasticity. Long-term depression refers to a long-lasting decrease in the efficacy and strength of synaptic transmission. Several mechanistically distinct forms of this type of synaptic plasticity have been identified, and the key step is believed to be the endocytosis of AMPARs into acidic organelles, such as endosomes and lysosomes (*5*, *15*). Therefore, an AMPAR probe with pH sensitivity will likely be useful for analyzing the internalization of endogenous AMPARs.

To date, two mechanisms have been proposed for the accumulation of AMPARs on the postsynaptic surface during LTP. The first is exocytosis from intracellular endosomes at extrasynaptic, peri-synaptic, or synaptic regions (*44*–*46*). The second is long-range lateral diffusion on the cell surface from the dendrites or extrasynaptic regions (*11*, *47*, *48*). However, it has remained controversial whether exocytosis or lateral diffusion is the main mechanism, mainly because of a lack of appropriate methods for evaluating the trafficking of AMPARs endogenously expressed in neurons without perturbing the receptors or neuronal function. To visualize AMPAR trafficking, SEP-tagged AMPARs on the N terminus (SEP-AMPARs) have been frequently used. SEP fluorescence is weakened under acidic conditions, which enables the visualization of exocytosis steps from acidic endosomes. However, although SEP-AMPARs are potent, they are overexpressed in most cases, which raises concerns that AMPAR distribution is affected. In addition, posttranslational modifications or associations with accessory proteins may be affected. It has also been suggested that fluorescence changes in SEP during LTP are caused by changes in intracellular pH rather than AMPAR localization changes (*51*). In contrast to previous techniques, our method was able to visualize the trafficking changes of endogenous AMPARs during LTP with minimal perturbation. We therefore believe that the results of the current study, in which exocytosis was identified as the main mechanism, are highly reliable. Given its relatively simple procedures, this technique is expected to be widely applied to other neuronal cultures, including brain slices, to clarify the relationship between AMPAR trafficking changes and neuronal functions.

## MATERIALS AND METHODS

### Synthesis

All synthesis procedures and compound characterizations are described in the Supplementary Materials.

### Construction of expression plasmids

cDNAs of mouse GluA1^flip^(Q), 5′UTR-GluA1^flip^(Q)-3′UTR, GluA2^flip^(Q), GluA2^flip^(Q)-3′UTR, GluA3^flip^(Q), GluA4^flip^(Q), GSG1L, and TARP γ7 were amplified from mouse brain marathon-ready cDNA (Clontech) by polymerase chain reaction (PCR). AMPARs cDNAs were subcloned into the expression vector, pCAGGS (kindly provided by J. Miyazaki, Osaka University, Osaka, Japan) or pCDM and auxiliary subunit cDNAs were subcloned into pIRES2-mCherry-F vector to obtain expression vectors. The pCDM vector was prepared from pcDNA3.1(+) (Invitrogen), in which the neomycin cassette was excised using *Pvu*II. 5′UTR-GluA1^flip^(R)-3′UTR, GluA2^flip^(R)-3′UTR, and GluA3^flip^(Q) (Y454A/R461G) (*52*) were obtained using the Q5 Site-Directed Mutagenesis Kit (NEB). To obtain Halo-tag-GluA2^flip^(Q)-3′UTR, cDNA encoding Halo-tag was amplified from the pFN21A Halo-tag CMV Flexi Vector (Promega). The Halo-tag cDNA was inserted into the pCAGGS expression vector before GluA2^flip^(Q)-3′UTR. cDNAs of rat GluK2(Q) was subcloned into the pCAGGS vector, and 2xHA-tag was inserted into downstream of the signal peptide of rat GluK2(Q).

The sequence of genetically engineered LBD named S1S2J was designed as the residues S383-K506 and P632-S775 of matured GluA2 conjugated through Gly and Thr as a linker, as previously reported (*29*). Besides, the three mutations G389R, L390G, and E391A were included. This gene fragment encoding the S1S2J was cloned into the modified T7 expression vector (*53*). The expression construct contained the additional Met and Gly to its N terminus, whereas the hexa-histidine tag was not included at the N terminus. All PCR-amplified DNAs were confirmed by DNA sequence analyses.

### Cell culture and transfection

HEK293T cells (American Type Culture Collection) were cultured in Dulbecco’s modified Eagle’s medium (DMEM)–GlutaMAX (Invitrogen) supplemented with 10% fetal bovine serum (FBS) (Sigma-Aldrich), penicillin (100 U ml^−1^), and streptomycin (100 μg ml^−1^) and incubated in a 5% CO_2_ humidified chamber at 37°C. Cells were transfected with a plasmid using Lipofectamine 3000 (Invitrogen) according to the manufacturer’s instructions. Twenty-four hours after transfection, the cells were seeded on glass-bottom dishes coated with poly-l-lysine solution and incubated for 24 hours except for GluA4, TARP γ7, and GluK2. For GluA4, TARP γ7, and GluK2 expression, the cells were used 24 hours after transfection. In the case of TARP γ7, 100 μM GYKI52466 was added to the dishes to prevent cation influx induced cytotoxicity after the transfection.

### Confocal live cell imaging of cell-surface AMPARs in HEK293T cells

HEK293T cells were cotransfected with plasmids for AMPAR [5′UTR-GluA1^flip^(R)-3′UTR, GluA2^flip^(Q)-3′UTR, GluA3^flip^(Q) (Y454A/R461G), and GluA4^flip^(Q)], kainate receptor [GluK2(Q)], auxiliary subunits, or the control vector and transfection marker (EFGP-F or mCherry-F). Fluorophore-ligand conjugates were diluted in Hepes-buffered saline (HBS) buffer [20 mM Hepes (pH 7.4), 107 mM NaCl, 6 mM KCl, 1.2 mM MgSO_4_, 2 mM CaCl_2_, and 11.5 mM glucose] and added to the cells. Confocal live imaging was performed with LSM900 (Carl Zeiss) equipped with a 63×, numerical aperture (NA) = 1.4 oil-immersion objective. Fluorescence images were acquired by excitation at 405, 488, 561, or 640 nm derived from diode lasers.

For competitive inhibition assay by NBQX, the stained cells were scanned first, and then 10 μM NBQX was added to the dish to inhibit probe binding.

For evaluating the concentration dependency of fluorophore-ligand conjugates, 0.1 nM probe in HBS was added to the dish. Then, probes in HBS were added stepwise to the dish to a final concentration of 0.3, 1, 3, 10, 30, 100, 300, 1000, and 3000 nM. Cell surface fluorescence intensity was quantified from the line scans of transfection marker positive cells and calculated with subtraction of background by ZEN blue software (Carl Zeiss). The membrane intensity was fitted with KaleidaGraph (Synergy software) to calculate the equilibrium constant value using the following equation: *a* + (*b* − *a*)/(1 + (*x*/*c*)^*d*). The equilibrium constant is regarded as the *K*_d_ in this study.

For Halo-tag labeling, HEK293T cells were transfected with Halo-tag-GluA2^flip^(Q)-3′UTR. The cells were incubated with 100 nM Halo-tag ligand-Janelia Fluor 635 [HTL(JF635)] (Promega) for 15 min at room temperature. After washing three times with HBS, 100 nM PFQX1(AF488) was added to the dishes.

For hemagglutinin (HA) staining, cells were treated with rabbit anti-HA tag antibody Alexa Fluor 647 conjugated (CST, 37297S, 1:400) for 15 min at room temperature.

### Expression and purification of S1S2J

The expression plasmids were transferred into *Escherichia coli* BL21(DE3) expression host cells. The cells were initially grown in 5 mL of LB medium supplemented with kanamycin (50 μg/ml) at 37°C for a day. An 800-μl volume of the cultured cells was then transferred to 800 ml of LB medium supplemented with kanamycin (50 μg/ml). The cells were cultured at 37°C until the OD_600_ (optical density at 600 nm) reached 0.6. At this point, 1 mM IPTG (isopropyl-β-d-thiogalactopyranoside) was added to the medium for expression of S1S2J, and the cells were cultured at 37°C for an additional 2 hours. The cells were harvested by centrifugation and stored at −80°C until use.

The cells were resuspended to 10 ml of buffer A [50 mM tris-HCl (pH 8.0), 100 mM NaCl, 1 mM EDTA, 1 mM phenylmethylsulfonyl fluoride (PMSF), 3 mM sodium deoxycholate, lysozyme (1 mg/ml), and DNase I (40 μg/ml)] and disrupted by sonication. After 15-min incubation at 4°C, the suspension was centrifuged at 17,000*g*, and the insoluble fraction was collected as the crude inclusion bodies. As followed by the same centrifugal separation, the crude inclusion bodies were washed with 20 ml of buffer B [50 mM tris-HCl (pH 8.0), 100 mM NaCl, 10 mM EDTA, 1 mM PMSF, and 0.5% (v/v) Triton X-100] and then buffer C [20 mM tris-HCl (pH 7.4), 200 mM NaCl, 1 mM EDTA, and 1 mM PMSF]. The prepared inclusion bodies were stored at −80°C until use.

The inclusion bodies were solubilized to 15 ml of buffer D [50 mM tris-HCl (pH 7.4), 5 mM EDTA, 8 M guanidine hydrochloride, and 50 mM dithiothreitol (DTT)] by stirring at room temperature for 4 hours. Then, 100 ml of cold buffer E [20 mM sodium acetate (pH 4.5), 1 mM EDTA, 4 M guanidine hydrochloride, and 1 mM DTT] was added to the denatured proteins and stirred for 1 hour at 4°C. The supernatant was collected after centrifugation at 17,000*g* for 1 hour, and the protein concentration was adjusted to 1.0 to 1.5 mg/ml with buffer E. This solution was dialyzed against 2 liters of buffer F [10 mM NaCl, 0.4 mM KCl, 650 mM arginine hydrochloride, and 1 mM EDTA (pH 8.5)] at 4°C for 16 hours (two times). The solubilized proteins were centrifuged at 17,000*g* for 1 hour, and the supernatant was collected. Then, 1 mM reduced glutathione and 0.2 mM oxidized glutathione were added to the supernatant and incubated at 4°C for a day. The solution was centrifuged at 17,000*g* for 1 hour, and the supernatant was collected. The supernatant was dialyzed against 2 liters of three different buffers in the following order: the buffer containing buffer F and buffer G [10 mM Hepes-NaOH (pH 7.4), 200 mM NaCl, and 1 mM EDTA] at a volume ratio of 1:1 at 4°C for 4 hours, the buffer containing buffer F and buffer G at a volume ratio of 1:9 at 4°C for 4 hours, and buffer G at 4°C for a day. After dialysis, the supernatant was separated from the precipitant by centrifugation at 17,000*g* for 1 hour. The supernatant was concentrated using Centricon Plus-70 with a 30-kDa cutoff (Millipore). The concentrated fraction was loaded onto HiLoad 26/600 Superdex 75 column (Cytiva) equilibrated with buffer G. After collecting the eluate containing the S1S2J, the buffer was exchanged to buffer H [10 mM Hepes-NaOH (pH 7.0), 20 mM NaCl, and 1 mM EDTA) by repetitive centrifugal concentration and dilution using Amicon Ultra 4 with a 30-kDa cutoff and concentrated to 10 mg/ml.

### Crystallization and x-ray diffraction data collection

To prepare the S1S2J bound to the ligand PFQX1(AF488), the purified S1S2J was mixed with 1 mM PFQX1(AF488) at 4°C for a day. The crystallization was performed by sitting-drop vapor-diffusion method using the JCSG-plus Eco screen (Molecular Dimensions). A crystallization robot, Gryphon (Art Robins Instruments), was used to mix 0.3 μl of the purified S1S2J with an equal volume of reservoir solution. The single crystal of S1S2J colored in yellow grew to dimensions of 0.7 mm by 0.2 mm by 0.2 mm in the reservoir [0.2 M lithium sulfate, 0.1 M tris-HCl (pH 8.5), and 40% (v/v) PEG-400 (polyethylene glycol, molecular weight 400)] within a day at 4°C. The crystal was frozen with liquid nitrogen. Diffraction data were collected on beamline BL45XU at SPring-8 (Hyogo, Japan) and automatically processed using the ZOO system (*54*). Data statistics are summarized in table S2.

### Structure determination and refinement

The structure of S1S2J bound to PFQX1(AF488) was determined by the molecular replacement (MR) method with AutoMR from the Phenix program package (*55*). The structure of the S1S2J in complex with an antagonist, NBQX (Protein Data Bank code: 6FQH), was used as the search model. Rotation and translation functions were calculated using the data of 47.3- to 1.8-Å resolution. Subsequent cycles of refinement were conducted using phenix.refine from the Phenix suite, alternating with manual fitting and rebuilding of the macromolecules, ions, polymers, and waters based on 2*F*_O_ − *F*_C_ and *F*_O_ − *F*_C_ electron density maps using Coot (*56*). Then, PFQX1(AF488) was built based on *F*_O_ − *F*_C_ electron densities and validated based on the polder omit map (*57*). The final refinement statistics and geometry, as defined by MolProbity (*58*), are presented in table S2. The structural figures were generated using PyMOL (http://pymol.org).

### Kinetics and reversibility of PFQX1(AF488) binding in HEK293T cells

HEK293T cells were transfected with Halo-tag-GluA2^flip^(Q)-3′UTR. The cells were incubated with 500 nM Halo-Tag ligand-Alexa647 [HTL(AF647)] for 15 min at room temperature. AF647 was used as a transfection marker. The cells were washed three times with HBS, and a cloning ring (16 mm φ) was placed on the dish to accelerate the diffusion of the probe solution. After the dish was set on the stage, 100 nM PFQX1(AF488) was added to inside the cloning ring and imaged at specified time points by confocal microscopy.

For evaluating reversibility, 100 nM PFQX1(AF488) in HBS was added to the dish. The cells were observed by confocal microscopy as the first image. Then, the cells were washed three times with HBS and scanned as the wash image. After removal of the buffer, 100 nM PFQX1(AF488) was re-treated, and the cells were scanned as the second image. To quantify the fluorescence intensity of the membrane, the average signal intensity of regions of interest (ROIs) set on the membrane was calculated after subtracting background fluorescence by ZEN blue software.

### Animals

Pregnant ICR (Institute of Cancer Research) mice maintained under specific pathogen–free conditions and C57BL/6J mice were purchased from Japan SLC Inc. (Shizuoka, Japan). The animals were housed in a controlled environment (23° ± 1°C, 12-hour/12-hour light/dark cycle) and had free access to food and water, according to the regulations of the Guidance for Proper Conduct of Animal Experiments by the Ministry of Education, Culture, Sports, Science, and Technology of Japan. All experimental procedures were performed in accordance with the National Institute of Health Guide for the Care and Use of Laboratory Animals and were approved by the Institutional Animal Use Committees of Nagoya University (reference nos. G230009 and G240012).

### Preparation of primary cultured hippocampal neurons

We modified the Banker’s culture method of hippocampal neurons (*59*). Glass-bottom dishes (14 mm φ, Matsunami) were coated with poly-d-lysine (Sigma-Aldrich) and washed three times with sterile dH_2_O. Hippocampi from 16-day-old ICR mouse embryos were aseptically dissected and digested with 0.25% (w/v) trypsin (Nacalai Tesque) for 20 min at 37°C. The cells were resuspended in Neurobasal Plus medium supplemented with 10% FBS, 1 mM GlutaMAX I (Invitrogen), penicillin (100 U/ml), and streptomycin (100 μg/ml) and filtered by Cell Strainer (100 μm, Falcon) followed by centrifugation at 1000 rpm for 5 min. The cells were resuspended in Neurobasal Plus medium supplemented with 2% of B-27 Plus Supplement, 1 mM GlutaMAX I (Invitrogen), penicillin (100 U/ml), and streptomycin (100 μg/ml) and plated at a density of 2 × 10^4^ cells on glass-bottom dishes. Precultured cortical astrocytes on glass coverslips (13 mm φ) were transferred to the dishes so that astrocytes were faced to neurons. The cultures were maintained at 37°C in a 95% air and 5% CO_2_ humidified incubator. The culture medium was replaced every 7 days, and the neurons were used at 16 to 18 days in vitro (DIV).

### Confocal live cell imaging of AMPARs in cultured neurons

Calcein Red AM (50 nM; AAT Bioquest) was added to the cultured neurons for 30 min at 37°C to visualize the intracellular region. The cells were washed with Hepes-based ACSF [25 mM Hepes (pH 7.4), 140 mM NaCl, 5 mM KCl, 1.3 mM CaCl_2_, 2 mM MgCl_2_, and 33 mM glucose], and 100 nM PFQX1(AF488) in Hepes-based ACSF was added to the dish.

For evaluating the concentration dependency of PFQX1(AF488), the cells were treated with 1 nM PFQX1(AF488). Then, PFQX1(AF488) in Hepes-based ACSF was added stepwise to the dish to a final concentration of 3, 10, 30, 100, 300, and 1000 nM. To quantify the concentration dependency after cLTP, the procedure described above was conducted after cLTP stimulation as described in the section “cLTP and quantification of cell-surface AMPARs.” The average intensity of ROIs set on spines was calculated by ZEN blue software after subtracting background fluorescence. The intensity was fitted with KaleidaGraph to calculate the equilibrium constant value using the following equation: *a* + (*b* − *a*)/(1 + (*x*/*c*)^*d*). The equilibrium constant is regarded as the *K*_d_ in this study.

### Kinetics and reversibility of PFQX1(AF488) binding in cultured neurons

A cloning ring (16 mm φ) was placed on the dish to accelerate the diffusion of the probe solution. After the dish was set on the stage, the cells were treated with 100 nM PFQX1(AF488) and imaged at specified time points by confocal microscopy.

For evaluating reversibility, stained cells were observed by confocal microscopy as the first image. Then, the cells were washed three times with Hepes-based ACSF and scanned as the wash image. After removal of the buffer, 100 nM PFQX1(AF488) was re-treated, and the cells were scanned as the second image. To quantify the fluorescence intensity of spines, the average signal intensity of ROIs set on spines was calculated by ZEN blue software after subtracting background fluorescence.

### cLTP and quantification of cell-surface AMPARs

After washing the cells with Hepes-based ACSF, 100 nM PFQX1(AF488) was added to the dish. The cells were scanned to obtain “basal state” images. Then, the cells were washed three times with cLTP stimulation buffer (Hepes-based ACSF except for MgCl_2_ supplemented with 20 μM bicuculline and 1 μM strychnine) to remove PFQX1(AF488). cLTP was induced by 200 μM glycine in cLTP stimulation buffer for 15 min at room temperature. After washing the cells with cLTP incubation buffer (Hepes-based ACSF supplemented with 20 μM bicuculline and 1 μM strychnine), the cells were incubated for 60 min at room temperature. Last, 100 nM PFQX1(AF488) was added to the dish, and images were scanned to obtain “after cLTP” images. Inhibitors (50 μM AP5 and 20 μM MK-801 for NMDARs and 5 μM KN93 for CaMKII and 25 μM bafilomycin A for exocytosis) were added to cLTP stimulation buffer and cLTP incubation buffer. To quantify the fluorescence intensity in spines, the average signal intensity of ROIs set on dendritic spines or dendritic shafts was calculated by ZEN blue software after subtracting background fluorescence. The fold change value was calculated using the following equation: (fluorescence intensity of “after cLTP” images)/(fluorescence intensity of “basal state” images).

### Two-step labeling and PFQX1(AF488) staining in cultured neurons

To label endogenous AMPARs, 10 μM CAM2(TCO) in the culture medium was gently added to the hippocampal neurons on a glass-bottom dish to a final concentration of 2 μM CAM2(TCO). The cells were incubated for 2 hours at 37°C. In the second step, the culture medium was removed, and the cells were treated with 100 nM Tz(ST647) for 5 min in Hepes-based ACSF at room temperature. To quench excess Tz(ST647), 1 μM TCO-OH in Hepes-based ACSF was added. Then, the cells were treated with 100 nM PFQX1(AF488). Imaging was performed with confocal microscopy.

### Statistical analysis and reproducibility

All data are expressed as means ± SEM. We accumulated the data for each condition from at least three independent experiments. We evaluated statistical significance with Student’s *t* test, one-way analysis of variance (ANOVA) with Dunnett’s test, or two-way ANOVA with Tukey’s or Sidak’s test. A value of *P* < 0.05 was considered significant.

Each experiment was repeated at least three times using independent biological samples.
