# Supplementary material for: Rapid and reversible fluorescent probe enables repeated snapshot imaging of AMPA receptors during synaptic plasticity
Source: Sci Adv. 2025 Jun 6;11(23):eadt6683. doi: 10.1126/sciadv.adt6683 (PMC12143348; doi:10.1126/sciadv.adt6683)

Science Advances
AAAS

Supplementary Materials for

Rapid and reversible fluorescent probe enables repeated snapshot imaging of AMPA receptors during synaptic plasticity

Kyohei Soga *et al.*

Corresponding author: Eriko Nango, eriko.nango.c4@tohoku.ac.jp;
Shigeki Kiyonaka, kiyonaka@chembio.nagoya-u.ac.jp

Sci. Adv. **11**, eadt6683 (2025)
DOI: 10.1126/sciadv.adt6683

This PDF file includes:

Supplementary Methods
Figs. S1 to S18
Tables S1 to S3

Supplementary Methods

Evaluation of the affinity of PFQX-amine

HEK293T cells were co-transfected with GluA2^{flip}(Q)-3'UTR and mCherry-F as a transfection marker. After washing the cells with HBS, 30 nM PFQX1(AF488) was added to the dish. Then, PFQX-amine in HBS was step-wisely added to the dish to a final concentration of 1, 3, 10, 30, 100, 300, 1,000, 3,000, 10,000 and 30,000 nM. Imaging was performed with confocal microscopy. Cell surface fluorescent intensity was quantified from the line scans of transfection marker positive cells calculated by *ZEN blue* software and calculated with subtraction of background. The membrane intensity was fitted with *KaleidaGraph* to calculate the IC_{50} value using the following equation: a + (b-a)/(1+(x/c)^d). The K_i value was calculated using the Cheng-Prusoff equation: $K_i = IC_{50}/(1+[S]/K_d)$. In the equation, [S] and K_d were the concentration and dissociation constant of PFQX1(AF488), respectively.

pH-dependency of fluorescent intensity

Fluorescent intensity was determined by spectrofluorophotometer RF-6000 (Shimadzu). PFQX1(Fl) or PFQX1(AF488) was added to the 0.1 M GTA buffer (0.1 M 3,3-dimethyl glutaric acid, 0.1 M Tris, 0.1 M 2-amino-2-methyl-1,3-propanediol) to a final concentration of 2 μM. pH value of GTA buffer was adjusted to 4, 5, 6, 7, 8, 9, and 10. Fluorescent intensity was normalized by the intensity at pH 10.

For the evaluation in cells, HEK293T cells expressing GluA2 or primary cultured hippocampal neurons were used. 100 nM PFQX1(AF488) was used and pH of extracellular solution (10 mM HEPES, 10 mM MES, 107 mM NaCl, 6 mM KCl, 1.2 mM $MgSO_4$, 2 mM $CaCl_2$, 11.5 mM glucose) was altered from 6.0 to 8.0.

Measurement of [Ca^{2+}]$_i$

HEK293T cells transfected with Ca^{2+}-permeable GluA1–4 (GluA1^{flip}(Q), GluA2^{flip}(Q), GluA3^{flip}(Q) (Y454A/R461G), and GluA4^{flip}(Q)) or GluK2(Q) were seeded on the 96 well plate. The cells were loaded with 5 µM Cal-520 AM (AAT Bioquest) in the culture medium for 2 h and washed with HBS using AquaMax 2000 (Molecular devices). The imaging experiment was carried out in an HBS buffer. For the fluorescence Ca^{2+} imaging, PFQX-amine was pretreated to the cells for 3 min followed by the addition of 30 µM glutamate with 100 µM cyclothiazide as a desensitization inhibitor for AMPARs and 100 µM kainic acid with 100 µM BPAM344 as a desensitization inhibitor for kainate receptors. Fluorescent intensity of Cal-520 was obtained using FlexStation3 (Molecular devices), and analyzed with SoftMax Pro 7 (Molecular devices). ΔRFU was defined as the difference between the maximum intensity value after adding glutamate and the average intensity value before adding the glutamate. ΔRFU was fitted with *KaleidaGraph*.

Immunostaining of cultured neurons

The cultured hippocampal neurons were fixed with cold methanol for 5 min and washed with PBS three times. Fixed cells were incubated with Blocking One Histo (Nacalai Tesque) for 10 min. The cells were washed with PBS containing 0.05% Tween 20 (PBS-T) once and incubated with primary antibodies in PBS-T containing 5% Blocking One Histo at room temperature for 2 h. Then, the cells were washed with PBS-T three times and incubated with secondary antibodies in PBS-T containing 5% Blocking One Histo for 1 h at room temperature. Following primary antibodies were used: mouse anti-PSD95 (abcam, ab2723, 1:1,000) or rabbit anti-MAP2 (Millipore, AB5622, 1:1,000). The

following secondary antibodies were used: donkey anti-mouse CF568 conjugated (Sigma, SAB4600075-250UL, 1:1,000), goat anti-mouse Alexa Fluor 647 conjugated (Invitrogen, A21235, 1:1,000), goat anti-rabbit Alexa Fluor 405 conjugated (abcam, ab175652, 1:1,000) and anti-rabbit Alexa Fluor Plus 488 conjugated (Invitrogen, A32731, 1,000). Imaging was performed with confocal microscopy.

With two-step labeling, the cells were labeled as described in "Two-step labeling and PFQX1(AF488) staining in cultured neurons," followed by immunostaining.

In fig. S9A, the cells were incubated with 500 nM CellTracker Red CMPTX (Invitrogen) instead of Calcein Red AM for 30 min at 37 ºC to visualize the intracellular region before the immunostaining.

Preparation and staining of cerebellar acute brain slices

C57BL/6J mice (3 weeks) were anesthetized with isoflurane, and their brains were isolated and immediately transferred to ice-cold cutting solution buffer (120 mM Choline Chloride, 3 mM KCl, 8 mM $MgCl_2$, 1.25 mM NaH_2PO_4, 28 mM $NaHCO_3$, 22 mM Glucose, and 0.5 mM Sodium ascorbate) gassed with 5% CO_2 and 95% O_2 and incubated for 5 min. Sagittal cerebellar slices were cut with a microslicer (Neo-linear slicer NLS-MT; DOSAKA EM) in cutting solution buffer and then incubated in ACSF (125 mM NaCl, 2.5 mM KCl, 2 mM $CaCl_2$, 1 mM $MgCl_2$, 1.25 mM NaH_2PO_4, 26 mM $NaHCO_3$, 10 mM Glucose) gassed with 5% CO_2 and 95% O_2 at 37 ºC.

Acute slices were transferred to glass bottom dishes and treated with 30 nM PFQX1(AF488) in HEPES-based ACSF for 5 min. Then, round coverslips were placed to prevent tissues from moving. Imaging was performed with confocal microscopy (LSM900).

Western blotting for quantification of AMPARs

cLTP was performed as described in "cLTP and quantification of cell-surface AMPARs". After washed with HBS, the cells were lysed with 100 µL of radio immunoprecipitation assay buffer (50 mM Tris-HCl pH 7.4, 150 mM NaCl, 1% NP-40, 0.5% sodium deoxycholate, 0.1% SDS, and 1 mM EDTA) containing 1% protease inhibitor cocktail (Nacalai Tesque) and incubated for 30 min at 4 °C on a mini rotary incubator. After mixing with 5 × Laemmli sample buffer (300 mM Tris-HCl pH 6.8, 15% SDS, 19.7% sucrose, and 0.05% bromophenol blue) containing 250 mM DTT, the samples were incubated for 30 min at room temperature with shaking. The samples were applied to SDS-PAGE (BIO-RAD Mini-Protean III) and electrotransferred onto an immuno-blot polyvinylidene fluoride membrane, followed by blocking with 5% nonfat dry milk in Tris-buffered saline (10 mM Tris-HCl pH 8.0, 150 mM NaCl) containing 0.05% Tween 20 (TBS-T). The membrane is incubated with primary antibodies in TBS-T supplemented with 1% nonfat dry milk overnight at 4 °C. Samples were detected by rabbit anti-GluA1 antibody (abcam, ab19491, 1:3,000), rabbit anti-GluA2 antibody (abcam, ab206293, 1:3,000), and rabbit βIII tubulin antibody (abcam, ab18207, 1:3,000). The membrane is washed three times with TBS-T and then treated with HRP-conjugated secondary antibody (MBL, 458, 1:3,000) in TBS-T supplemented with 1% nonfat dry milk for 1 h at room temperature. The membrane is washed three times with TBS-T. Chemiluminescent signals generated with ECL Start or ECL Prime (GE Healthcare) were detected with a Fusion Solo S imaging system (Vilber Lourmat). The band intensity of GluA1 and GluA2 was normalized by the intensity of βIII-tubulin.

Exocytosis assay of AMPARs in HEK293T cells

HEK293T cells expressing Halo-tag-GluA2^{flip}(Q)-3'UTR were treated with Halo-tag ligand-Alexa488 (HTL(AF488)) for 30 min at room temperature as a first pulse. After washing with DMEM three times, the cells were incubated with 25 μM brefeldin A or DMSO for 2 h at 37 °C in a 95% air and 5% CO_2 humidified incubator. Then, HTL(AF647) was added to the dishes and incubated for 15 min at room temperature as a second pulse to visualize spontaneous exocytosed AMPARs. Labeled cells were fixed with 4% PFA for 15 min. The fluorescent images were obtained using confocal microscopy (LSM900).

General materials and methods for organic synthesis

All chemical reagents and solvents were purchased from commercial sources (FUJIFILM Wako pure chemical, TCI chemical, Sigma-Aldrich, Click Chemistry Tools, BroadPharm) and were used without further purification. Thin-layer chromatography (TLC) was performed on silica gel 60 F254 precoated aluminum sheets (Merck). Chromatographic purification was performed by flash column chromatography on silica gel 60 N (neutral, 40–50 μm, Kanto Chemical). ^{1}H-NMR spectra were recorded in deuterated solvents on an Avance III HD 300 MHz or 500 MHz (Bruker). Chemical shifts were referenced to residual solvent peaks or tetramethyl silane (δ = 0 ppm). Multiplicities are abbreviated as follows: s = singlet, d = doublet, dd = double doublet, t = triplet, m = multiplet. High-resolution mass spectra were measured on a Compact (Bruker) equipped with electron spray ionization (ESI). Reversed-phase HPLC (RP-HPLC) was performed on a Hitachi Chromaster system equipped with a diode array and a YMC-Pack ODS-A column.

Synthesis of PFQX1(Fl)

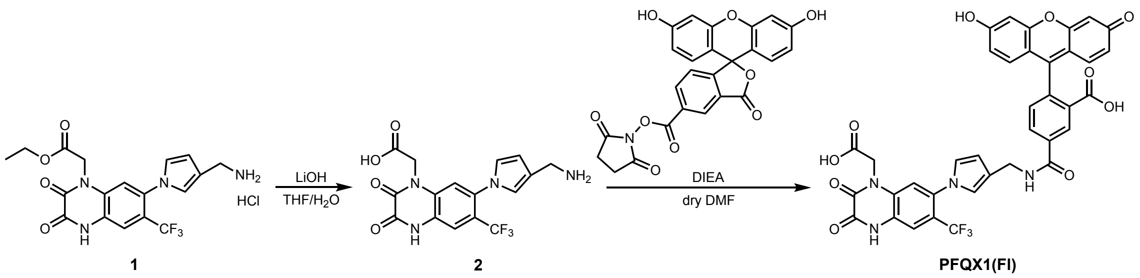

Synthesis of compound **2 (PFQX-amine)**

A solution of **1** (*20*) (64 mg, 0.14 mmol) and $LiOH{\cdot}H_2O$ (12 mg, 0.28 mmol) in THF (0.5 mL) and H_2O (0.5 mL) was stirred for 16 h at room temperature. After neutralization of the solvent with 1 M HCl, the crude was passed through a short pad of silica gel to give white crude **2** (76 mg) and used for the next step without further purification. A small portion of the crude was purified by RP-HPLC for the biological assay. The HPLC condition was as follows; (ODS-A, 250 × 20 mm, mobile phase; CH_3CN (containing 0.1% TFA) : H_2O (containing 0.1% TFA) = 10 : 90 → 30 : 70 (linear gradient over 20 min), flow rate; 10 mL/min, detection; UV (250 nm)). ^{1}H-NMR (300 MHz, CD_3OD) δ 7.52 (s, 1H), 7.13 (s, 1H), 6.94 (s, 1H), 6.82 (s, 1H), 6.30-6.28 (m, 1H), 4.87 (s, 2H), 3.94 (s, 2H). HR-ESI MS m/z calcd for $[M+Na]^+$ 405.0781, found 405.0762.

Synthesis of **PFQX1(Fl)**

A solution of crude **2** (1.8 mg), DIEA (4.4 µL, 25 µmol), and 5-carboxyfluorescein NHS ester (1.0 mg, 2.1 µmol) in dry DMF (1 mL) was stirred for 15.5 h at room temperature under a nitrogen atmosphere. The crude mixture was purified by RP-HPLC (ODS-A, 250 × 10 mm, mobile phase; CH_3CN (containing 0.1% TFA) : H_2O (containing 0.1% TFA) = 10 : 90 → 50 : 50 (linear gradient over 40 min), flow rate; 3 mL/min, detection; UV (250

nm)) to give **PFQX1(Fl)** (0.97 mg, 1.3 μmol, 62% yield) as an orange solid. ^{1}H-NMR (500 MHz, CD_3OD) δ 8.54 (s, 1H), 8.25 (dd, *J* = 1.5, 8.5 Hz, 1H), 7.61 (s, 1H), 7.38 (d, *J* = 8.0 Hz, 1H), 7.29 (s, 1H), 6.92 (s, 1H), 6.86-6.83 (m, 5H), 6.71 (d, *J* = 8.0 Hz, 2H), 6.35 (s, 1H), 5.02 (s, 2H), 4.54 (s, 2H). HR-ESI MS m/z calcd for $[M-H]^-$ 739.1294, found 739.1305.

Synthesis of PFQX2(Fl)

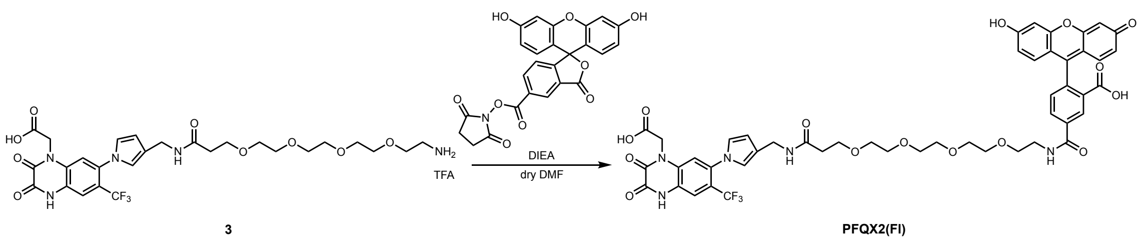

A solution of **3** (*20*) (4.0 mg), DIEA (10 µL, 57 µmol), and 5-carboxyfluorescein NHS ester (1.0 mg, 2.1 µmol) in dry DMF (1 mL) was stirred for 15.5 h at room temperature under a nitrogen atmosphere. The crude mixture was purified by RP-HPLC (ODS-A, 250 × 10 mm, mobile phase; CH_3CN (containing 0.1% TFA) : H_2O (containing 0.1% TFA) = 10 : 90 → 60 : 40 (linear gradient over 50 min), flow rate; 3 mL/min, detection; UV (250 nm)) to give **PFQX-L-Fl** (1.3 mg, 1.3 µmol, 62% yield) as an orange solid. ^{1}H-NMR (500 MHz, CD_3OD) δ 8.54 (s, 1H), 8.24 (dd, J = 1.5, 8.0 Hz, 1H), 7.59 (s, 1H), 7.37 (d, J = 8.0 Hz, 1H), 7.24 (s, 1H), 6.87-6.71 (m, 8H), 6.21 (s, 1H), 4.98 (s, 2H), 4.26 (s, 2H), 3.72-3.54 (m, 18H), 2.45 (t, J = 6.0 Hz, 2H). HR-ESI MS m/z calcd for $[M-H]^-$ 986.2713, found 986.2690.

Synthesis of PFQX1(TAMRA)

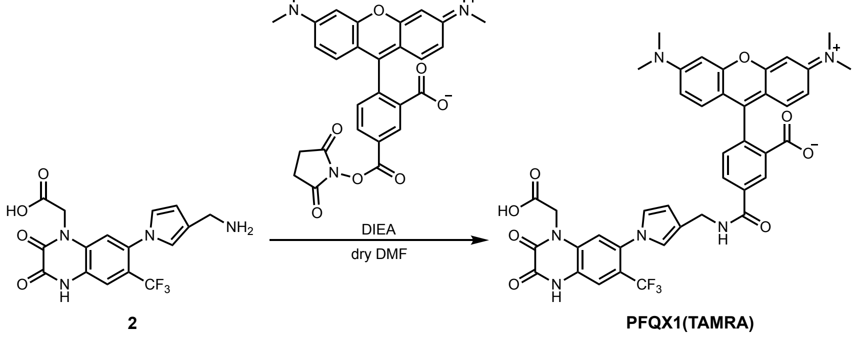

A solution of crude **2** (1.5 mg), DIEA (1.6 µL, 10 µmol), and 5-TAMRA NHS ester (1.0

mg, 1.9 μmol) in dry DMF (1 mL) was stirred for 16 h at room temperature under a nitrogen atmosphere. The crude mixture was purified by RP-HPLC (ODS-A, 250 × 10 mm, mobile phase; CH_3CN : 10 mM $AcONH_4$ aq. = 10 : 90 → 70 : 30 (linear gradient over 60 min), flow rate; 3 mL/min, detection; UV (250 nm)) to give **PFQX1(TAMRA)** (0.96 mg, 1.2 μmol, 63% yield) as a pink solid. ^{1}H-NMR (300 MHz, CD_3OD) δ 8.65 (s, 1H), 8.16 (dd, J = 7.8 Hz, 1H), 7.58 (s, 1H), 7.43 (d, J = 7.8 Hz, 1H), 7.22 (m, 3H), 7.05 (dd, J = 9.6, 2.4 Hz, 2H), 6.93 (m, 3H), 6.82 (s, 1H), 6.34 (t, J = 1.2 Hz, 1H), 4.86 (s, 2H), 4.54 (s, 2H), 3.29 (s, 12H). HR-ESI MS m/z calcd for $[M+H]^+$ 795.2385, found 795.2386.

Synthesis of PFQX1(AF488)

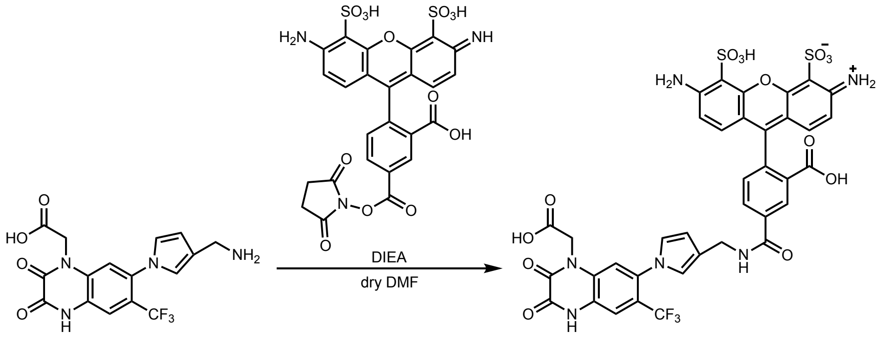

A solution of crude **2** (1.3 mg), DIEA (1.6 μL, 10 μmol), and Az488 NHS ester (1.0 mg, 1.6 μmol) in dry DMF (1 mL) was stirred for 16 h at room temperature under a nitrogen atmosphere. The crude mixture was purified by RP-HPLC (ODS-A, 250 × 10 mm, mobile phase; CH_3CN : 10 mM $AcONH_4$ aq. = 0 : 100 → 40 : 60 (linear gradient over 40 min), flow rate; 3 mL/min, detection; UV (250 nm)) to give **PFQX1(AF488)** (1.0 mg, 1.1 μmol, 69% yield) as an orange solid. ^{1}H-NMR (300 MHz, D_2O) δ 8.29 (s, 1H), 7.97 (dd, J = 1.4, 7.9 Hz, 1H), 7.64 (s, 1H), 7.34 (d, J = 8.0 Hz, 1H), 7.27 (s, 1H), 7.17 (d, J = 9.3 Hz, 2H), 7.01 (s, 1H), 6.94 (s, 1H), 6.90 (d, J = 9.3 Hz, 2H), 6.38 (t, J = 2.2 Hz, 1H), 4.73 (s, 2H), 4.52 (s, 2H). HR-ESI MS m/z calcd for $[M-2H+Na]^-$ 919.0569, found 919.0588.

Synthesis of HTL(AF647)

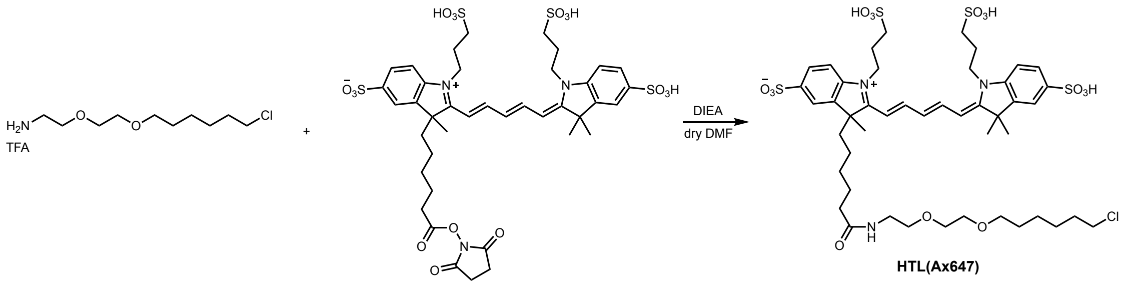

2-(2-((6-chlorohexyl)oxy)ethoxy)ethanamine TFA salt was synthesized as described in (*13*). A solution of 2-(2-((6-chlorohexyl)oxy)ethoxy)ethanamine TFA salt (0.70 mg, 2.1 μmol), DIEA (1.0 μL, 6.0 μmol), and Alexa Fluor 647 NHS ester (1.0 mg, 1.0 μmol) in dry DMF (1 mL) was stirred for 19 h at room temperature under a nitrogen atmosphere. The crude mixture was purified by RP-HPLC (ODS-A, 250 × 10 mm, mobile phase; CH_3CN : 10 mM $AcONH_4$ aq. = 5 : 95 → 55 : 45 (linear gradient over 50 min), flow rate; 3 mL/min, detection; UV (250 nm)) to give **HTL(AF647)** (0.83 mg, 0.77 μmol, 74% yield) as a blue solid. HR-ESI MS m/z calcd for $[M]^{\bullet+}$ 1063.3060, found 1063.3067.

Synthesis of HTL(AF488)

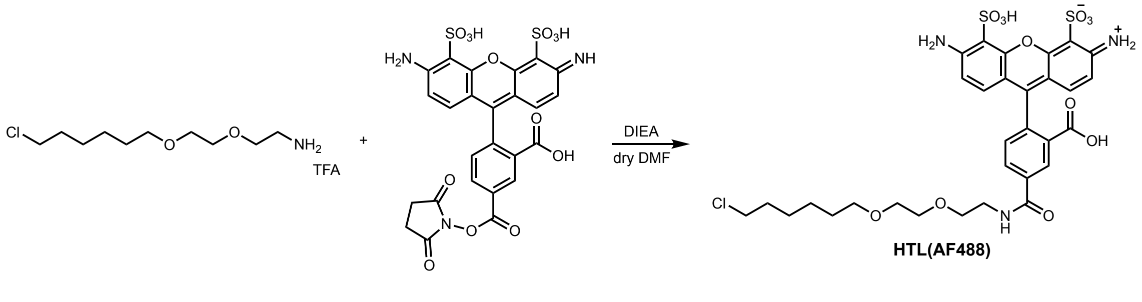

2-(2-((6-chlorohexyl)oxy)ethoxy)ethanamine TFA salt was synthesized as described in (*13*). A solution of 2-(2-((6-chlorohexyl)oxy)ethoxy)ethanamine TFA salt (0.9 mg, 2.4 μmol), DIEA (5.0 μL, 10.3 μmol), and Alexa Fluor 488 NHS ester (1.0 mg, 1.6 μmol) in dry DMF (1 mL) was stirred for 18 h at room temperature under a nitrogen atmosphere.

The crude mixture was purified by RP-HPLC (ODS-A, 250 × 10 mm, mobile phase; CH_3CN : 10 mM $AcONH_4$ aq. = 20 : 80 → 50 : 50 (linear gradient over 30 min), flow rate; 3 mL/min, detection; UV (250 nm)) to give **HTL(AF488)** (0.22 mg, 0.29 μmol, 18% yield) as an orange solid. HR-ESI MS m/z calcd for $[M-2H]^{2-}$ 369.5489, found 369.5544.

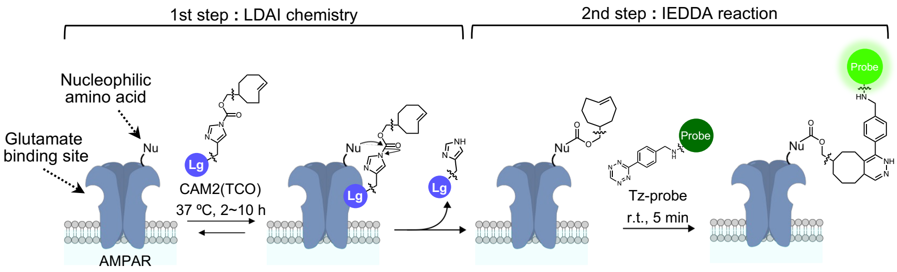

Fig. S1. Ligand-directed two-step labeling of AMPARs. A schematic illustration of ligand-directed two-step labeling. In the 1st step, TCO is covalently attached to AMPARs using CAM2(TCO) by ligand-directed acyl imidazole (LDAI) chemistry. In the 2nd step, tetrazine conjugated fluorescent dye (Tz-probe) is selectively tethered to TCO via inverse electron demand Diels-Alder (IEDDA) reaction. Cell-surface AMPARs can be selectively labeled with the dye because of the extremely high reaction rate of IEDDA reaction.

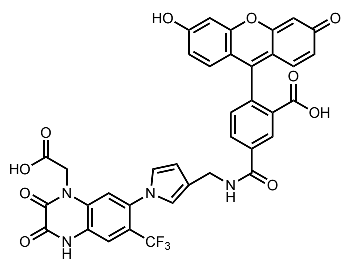

PFQX1(Fl)

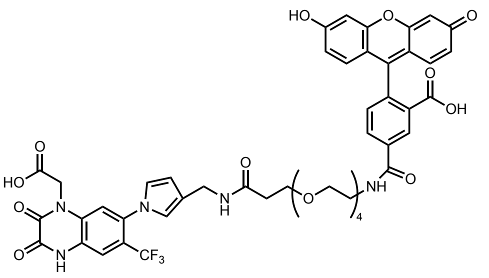

PFQX2(Fl)

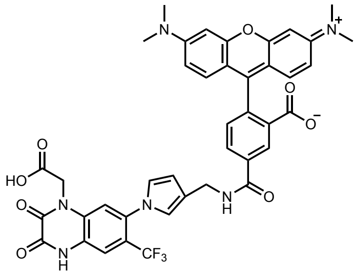

PFQX1(TAMRA)

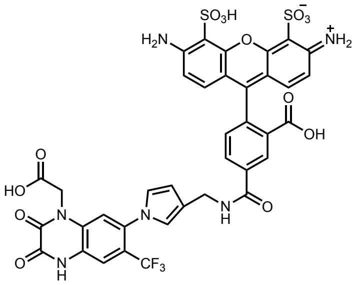

PFQX1(AF488)

Fig. S2. Detailed chemical structures of fluorophore-ligand conjugates. Chemical structures of PFQX1(Fl), PFQX2(Fl), PFQX1(TAMRA) and PFQX1(AF488) are shown.

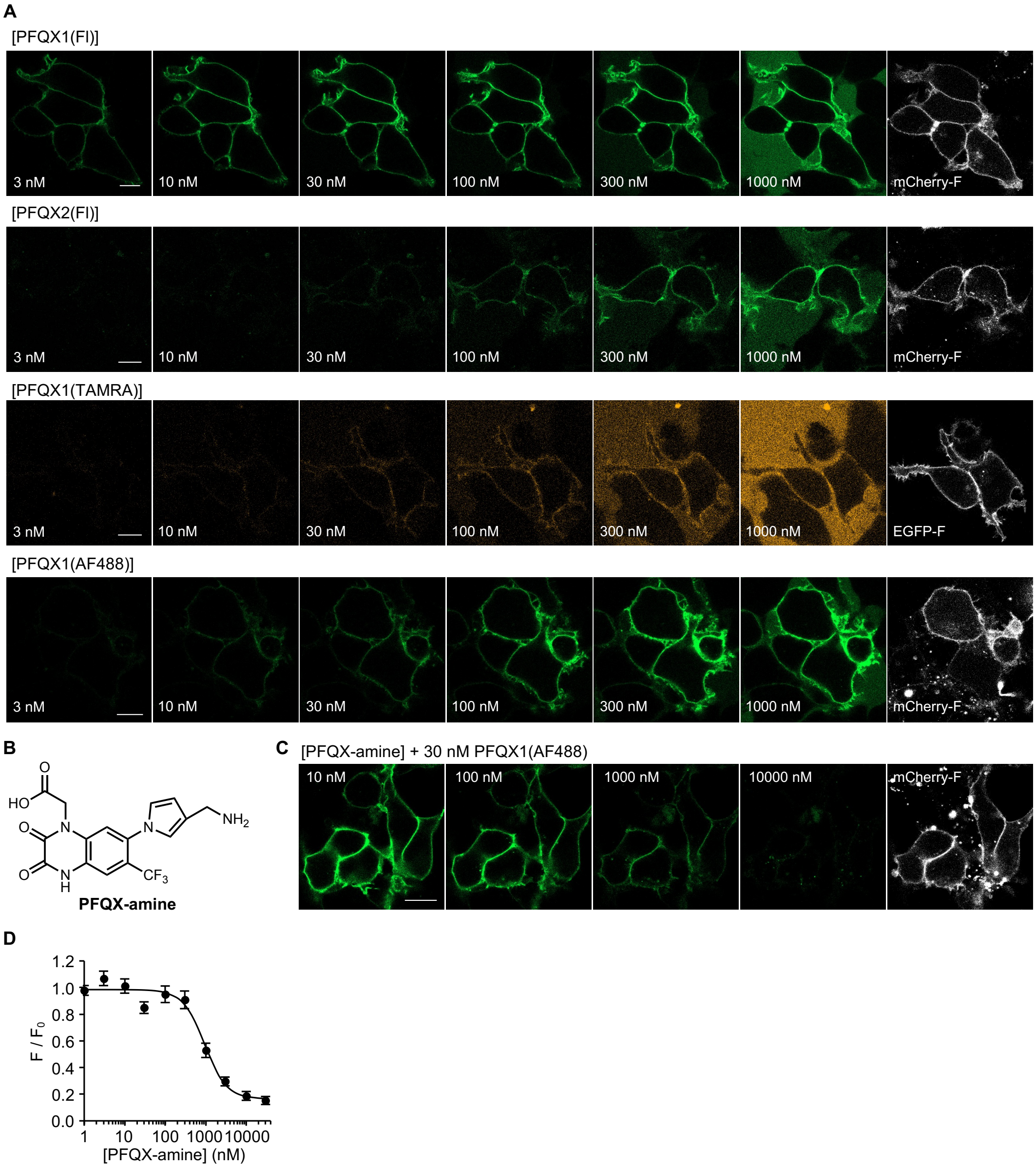

Fig. S3. Concentration-dependency of fluorophore-ligand conjugates and the affinity of PFQX-amine to AMPARs. (A) Concentration-dependent binding of fluorophore-ligand conjugates to GluA2. Representative results of confocal live cell imaging of HEK293T cells transfected with GluA2 are shown. mCherry-F (for Fl and AF488) and EGFP-F (for TAMRA) were used as transfection markers. Scale bars, 10 µm. **(B–D)** Competitive inhibition of PFQX1(AF488) binding by PFQX-amine. In **B**, chemical structure of PFQX-amine, the synthetic intermediate of fluorophore-ligand conjugates, is shown. In **C**, representative confocal live imaging of HEK293T cells transfected with GluA2 in the presence of each concentration of PFQX-amine are shown. mCherry-F was used as a transfection marker. In **D**, concentration-dependency of PFQX-

amine is shown. The surface intensity of PFQX1(AF488) was quantified, which was normalized to that of 0 nM PFQX-amine. The IC_{50} value was 862 ± 309 nM. The K_i value was 477 ± 171 nM, which was calculated using the Cheng-Prusoff equation. [PFQX1(AF488)] = 30 nM (n = 3). Data are represented as mean ± s.e.m.

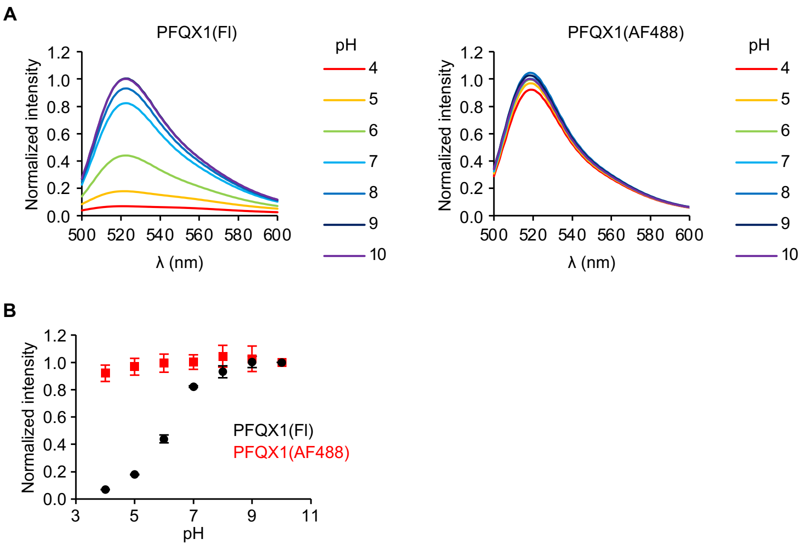

Fig. S4. pH-dependency of PFQX1(Fl) and PFQX1(AF488). (A) Fluorescence spectra of PFQX1(Fl) (left) and PFQX1(AF488) (right) are shown. The fluorescence intensity was normalized to that at pH 10. Samples were prepared in GTA buffer (pH4–10). **(B)** pH titration curve of PFQX1(Fl) and PFQX1(AF488). [PFQX1(Fl)] = 2 μM, [PFQX1(AF488)] = 2 μM (n = 3). λ_{ex} = 480 nm. Data are represented as mean ± s.e.m.

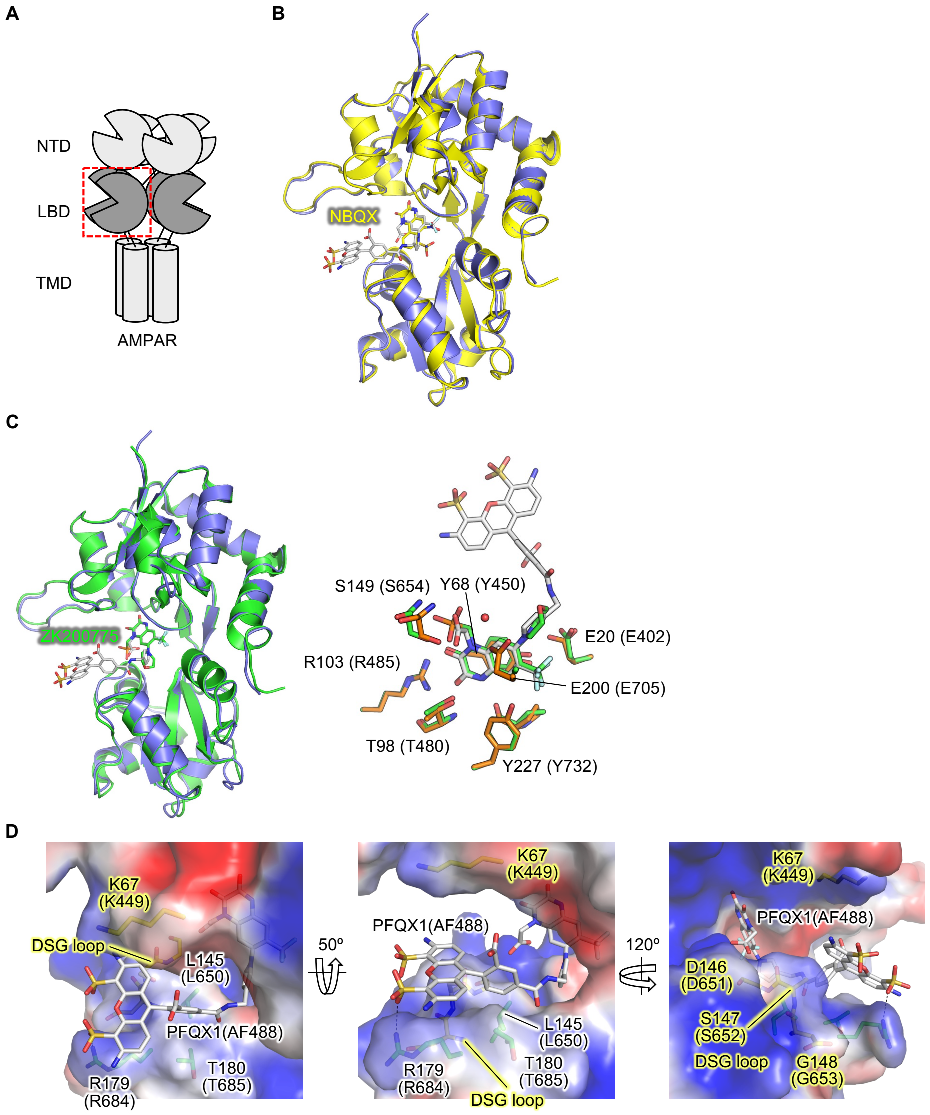

Fig. S5. Comparison of the X-ray structure of PFQX1(AF488) bound S1S2J with other antagonist bound forms. The residue numbers of S1S2J are shown, and the corresponding residue of full-length GluA2 is also shown in parentheses. **(A)** Schematic representation of AMPAR. Each subunit of AMPAR is composed of the amino-terminal domain (NTD), the ligand-binding domain (LBD), and the transmembrane domain (TMD). S1S2J is a recombinant protein derived from the LDB, as indicated by the red dot square. **(B)** Superposed structures of S1S2J bound to NBQX (PDB code: 6FQH) (yellow) and PFQX1(AF488) (purple). Bound NBQX and PFQX1(AF488) are shown as sticks. **(C)** Superposed structures of ligand binding domain bound to ZK200757 (PDB code: 5KBV) (green) and PFQX1(AF488) (purple). Bound ZK200757 and PFQX1(AF488) are shown as sticks. Right, close-up view of the ligand binding site. ZK200757 and the residues interacted with the ligand are shown as sticks (green). PFQX1(AF488) and the residues interacted with PFQX part of the ligand are also shown as sticks (white and orange, respectively). **(D)** The electrostatic potential around the

AF488 binding region of the S1S2J. The residues involved in recognizing the AF488 part are shown as sticks (green), while Lys67 (Lys449) and DSG loop are also shown as sticks (yellow).

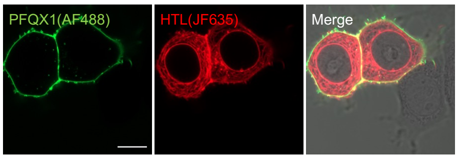

Fig. S6. Cell surface specificity of PFQX1(AF488) staining. HEK293T cells expressing Halo-tag fused GluA2 were treated with 100 nM cell-permeable Halo-tag probe, HTL(JF635), to stain cell surface and intracellular AMPARs. Then, 100 nM PFQX1(AF488) was added to cells. Scale bar, 10 µm.

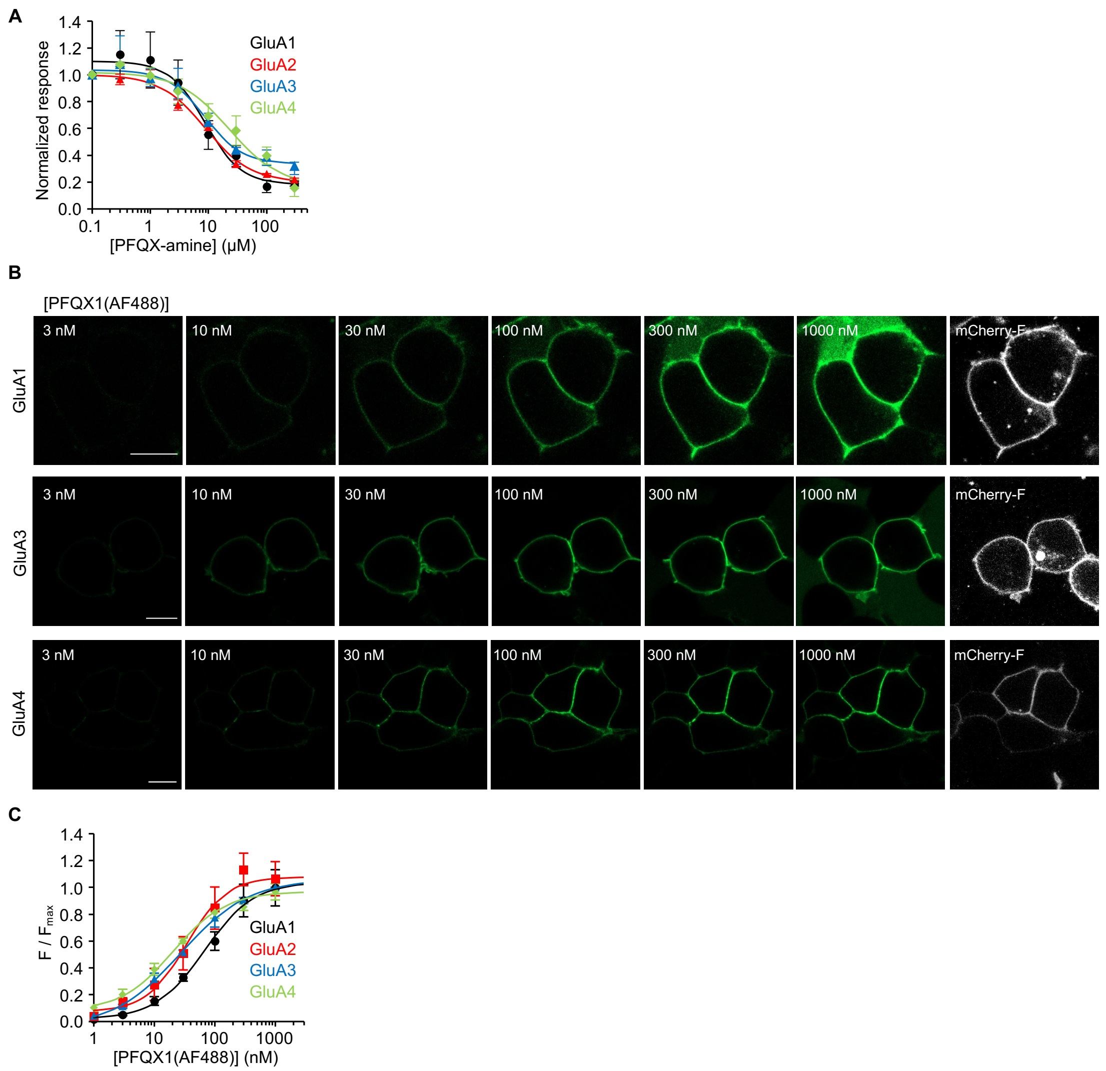

Fig. S7. Non-specific action of PFQX derivatives to AMPAR subunits. (A) Fluorescence Ca^{2+} assay to analyze the inhibition of 30 μM glutamate-induced response by PFQX-amine. HEK293T cells were transfected with GluA1, GluA2, GluA3 (Y454A/R461G), or GluA4. The mutation was introduced to GluA3 to enhance surface expression of the homomer. The intensity was normalized to that at 0.1 μM PFQX-amine (n = 3). **(B)** Representative results of confocal live cell imaging of HEK293T cells expressing GluA1, GluA3 (Y454A/R461G), and GluA4 are shown. mCherry-F was used as transfection markers. The cells were treated with PFQX1(AF488) at each concentration. Scale bars, 10 μm. **(C)** Surface intensity of PFQX1(AF488) was quantified (n = 3). The K_d values for GluA1, GluA3 and GluA4 were 78.7 ± 15.4 nM, 30.6 ± 7.0 nM and 20.2 ± 1.2 nM, respectively. Summarized data is shown in table S1. Data are represented as mean ± s.e.m.

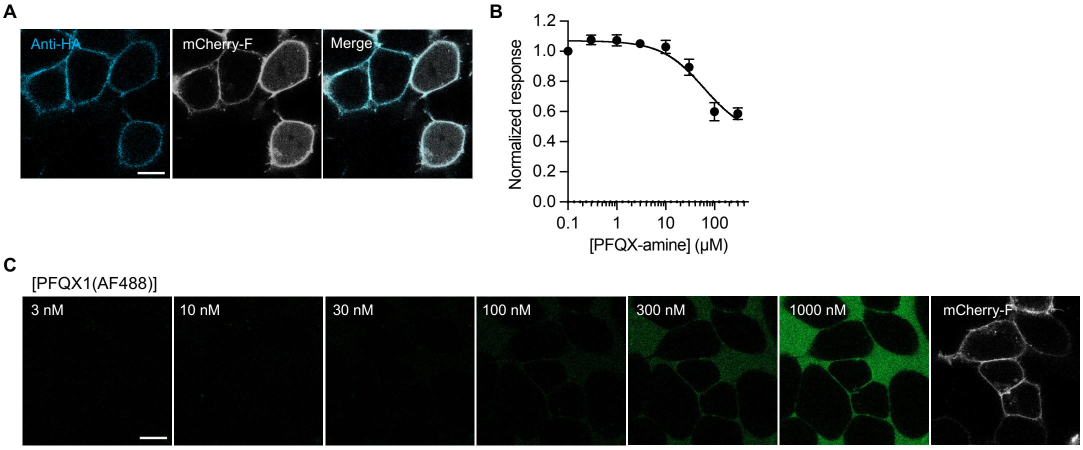

Fig. S8. Sub-micromolar concentrations of PFQX1(AF488) fail to visualize cell surface kainate receptors in HEK293T cells. (A) HA-tag staining of GluK2(Q). GluK2(Q) expressing cells were treated with anti-HA antibody (Alexa Fluor 647 conjugate) for 15 min. Scale bar, 10 μm. **(B)** Fluorescence Ca^{2+} assay to analyze the inhibition of 100 μM kainic acid-induced response by PFQX-amine. HEK293T cells were transfected with GluK2(Q). The intensity was normalized to that at 0.1 μM PFQX-amine (n = 3). **(C)** Representative results of confocal live cell imaging of HEK293T cells expressing GluK2(Q) are shown. mCherry-F was used as a transfection marker. Cells were treated with PFQX1(AF488) at each concentration. Scale bar, 10 μm. Data are represented as mean ± s.e.m.

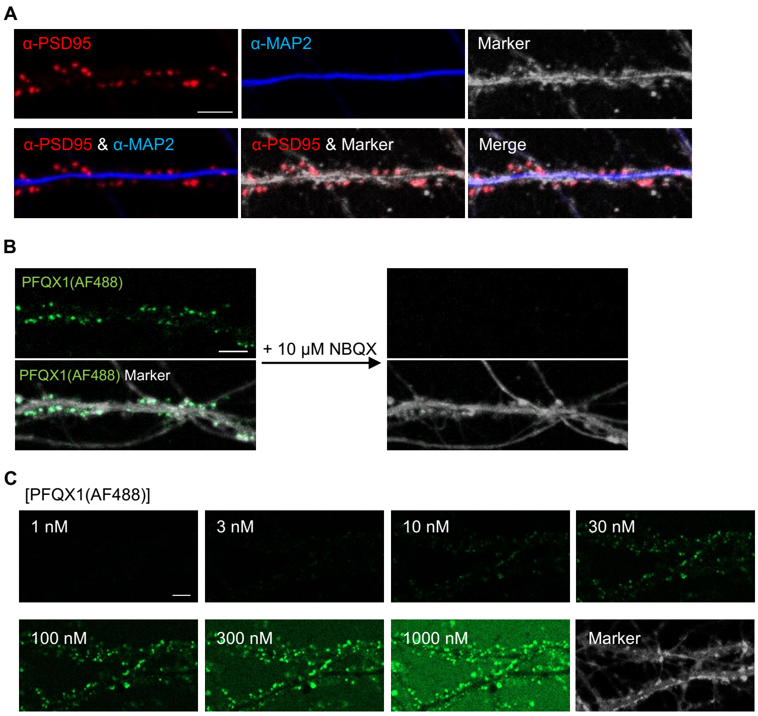

Fig. S9. Immunostaining and concentration-dependency of PFQX1(AF488) binding to AMPARs in cultured hippocampal neurons. (A) Immunostaining of cultured hippocampal neurons. The cells were immunostained with anti-PSD95 (red signal) and anti-MAP2 (blue signal) after staining with CellTracker Red CMPTX (gray signal). Scale bar, 5 µm. **(B)** Competitive inhibition of PFQX1(AF488) binding by NBQX. 100 nM PFQX1(AF488) was added to the cultured hippocampal neurons. Then, 10 µM NBQX was added to the medium. Scale bar, 5 µm. **(C)** Concentration-dependency of PFQX1(AF488) binding to AMPARs in cultured hippocampal neurons. Representative results of confocal live cell imaging are shown. Quantification data is shown in Fig. 4C. Scale bar, 5 µm.

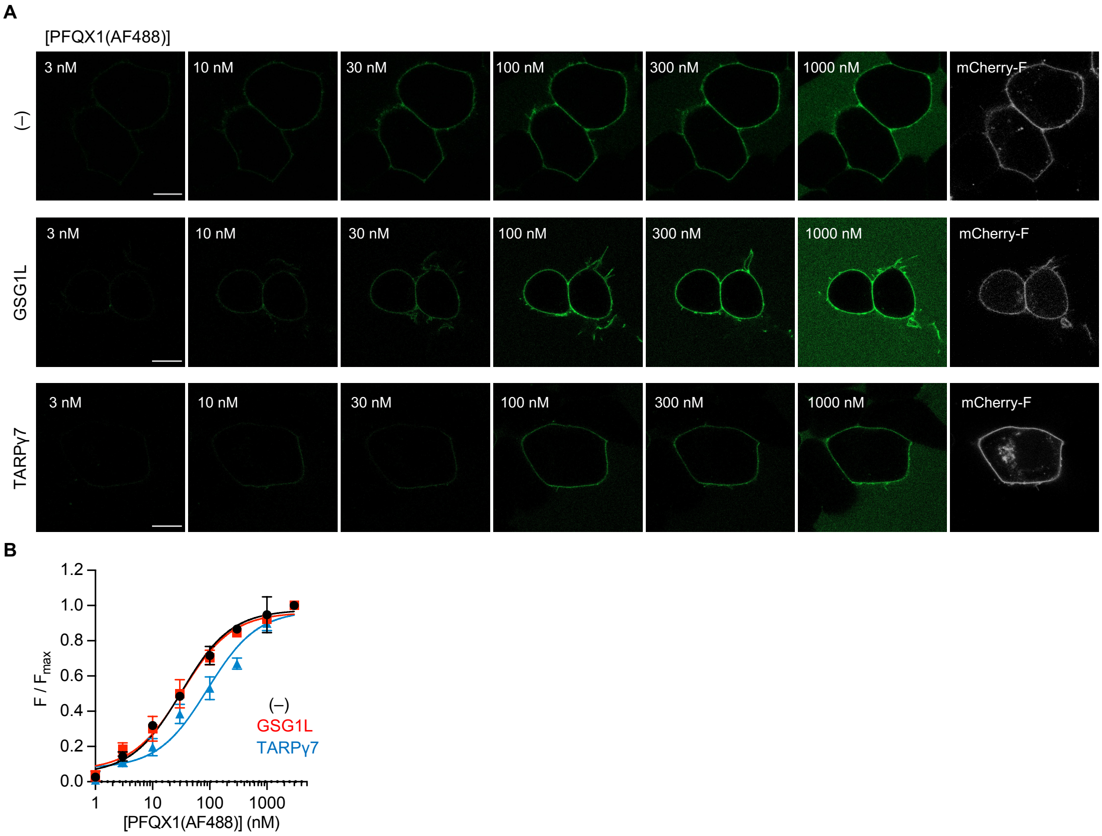

Fig. S10. The effect of auxiliary subunits on the PFQX1(AF488) affinity to AMPARs. (A) Concentration-dependent binding of PFQX1(AF488) to GluA2^{flip}(R) expressed with GSG1L or TARP γ7. GluA2^{flip}(R) was used to prevent calcium-influx induced cytotoxicity. Representative results of confocal live cell imaging are shown. mCherry-F was used as transfection markers. Scale bars, 10 µm. **(B)** The surface intensity of PFQX1(AF488) was quantified. The K_d values for GluA2^{flip}(R), expressed alone (–), with GSG1L, and with TARP γ7 were 32.3 ± 4.7 nM, 34.5 ± 12.4 nM, and 118.3 ± 44.7 nM, respectively (n = 3–4). Summarized data is shown in table S1. Data are represented as mean ± s.e.m.

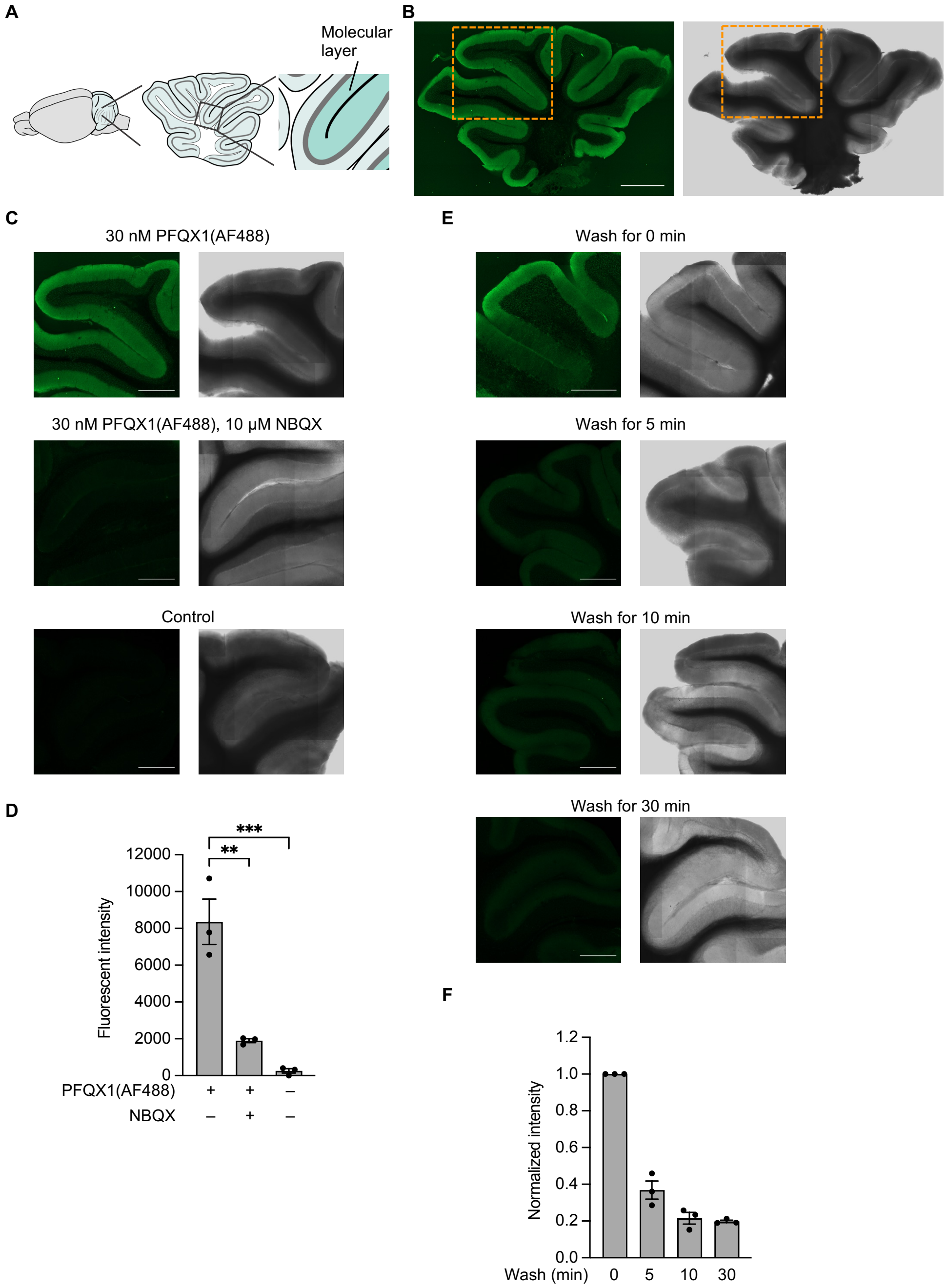

Fig. S11. AMPARs staining in acute cerebellar slices. (A) Schematic illustrations of cerebellum. AMPARs are selectively expressed in the molecular layer. **(B)** Confocal images of acute cerebellar slices treated with 30 nM PFQX1(AF488) for 5 min. The orange ROI is expanded in the upper image of **C**. Scale bar, 500 µm. **(C)** Competitive inhibition of PFQX1(AF488) binding by NBQX. Slices were simultaneously treated with 30 nM PFQX1(AF488) and 10 µM NBQX for 5 min. Scale bars, 500 µm. **(D)** Quantification of probe intensity in **C**. ROIs were blindly placed on cerebellar lobules and the background signal was subtracted from the white matter (n = 3). **(E)** Washout

properties of PFQX1(AF488) in brain tissues. Slices were treated with 30 nM PFQX1(AF488) for 5 min and washed twice with HEPES-based ACSF. Then, slices were incubated in HEPES-based ACSF with shaking on a rocking shaker for the indicated time. Scale bars, 500 μm. **(F)** Quantification of probe intensity in **E**. ROIs were blindly placed on cerebellar lobules and the background signal was subtracted from the white matter ($n = 3$). The intensity was normalized to that of the 0 min wash. Significant difference (*** $p < 0.001$, ** $p < 0.01$, One-way ANOVA with Dunnett's test). Data are represented as mean ± s.e.m.

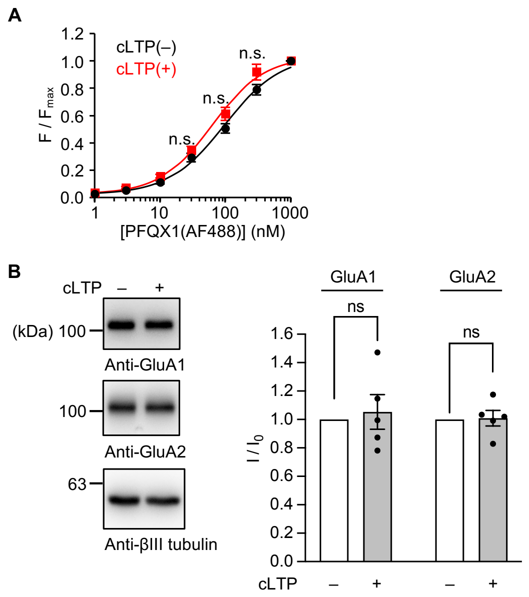

Fig. S12. Binding affinity to PFQX1(AF488) and expression level of AMPARs were not affected by cLTP. (A) Concentration-dependency of PFQX1(AF488) binding to AMPARs in cultured hippocampal neurons was evaluated before or after cLTP. The K_d value after cLTP was 72.4 ± 17.4 nM (n = 3). **(B)** Western blotting analysis of cultured hippocampal neurons. The expression levels of GluA1, GluA2, and βIII tubulin as a loading control were analyzed before and after cLTP stimulation. Left, representative blots are shown. Right, quantification of band intensity. Band intensity was normalized to cLTP(–) (n = 5). ns, not significant ($p > 0.05$, Student's t-test.). Data are represented as mean ± s.e.m.

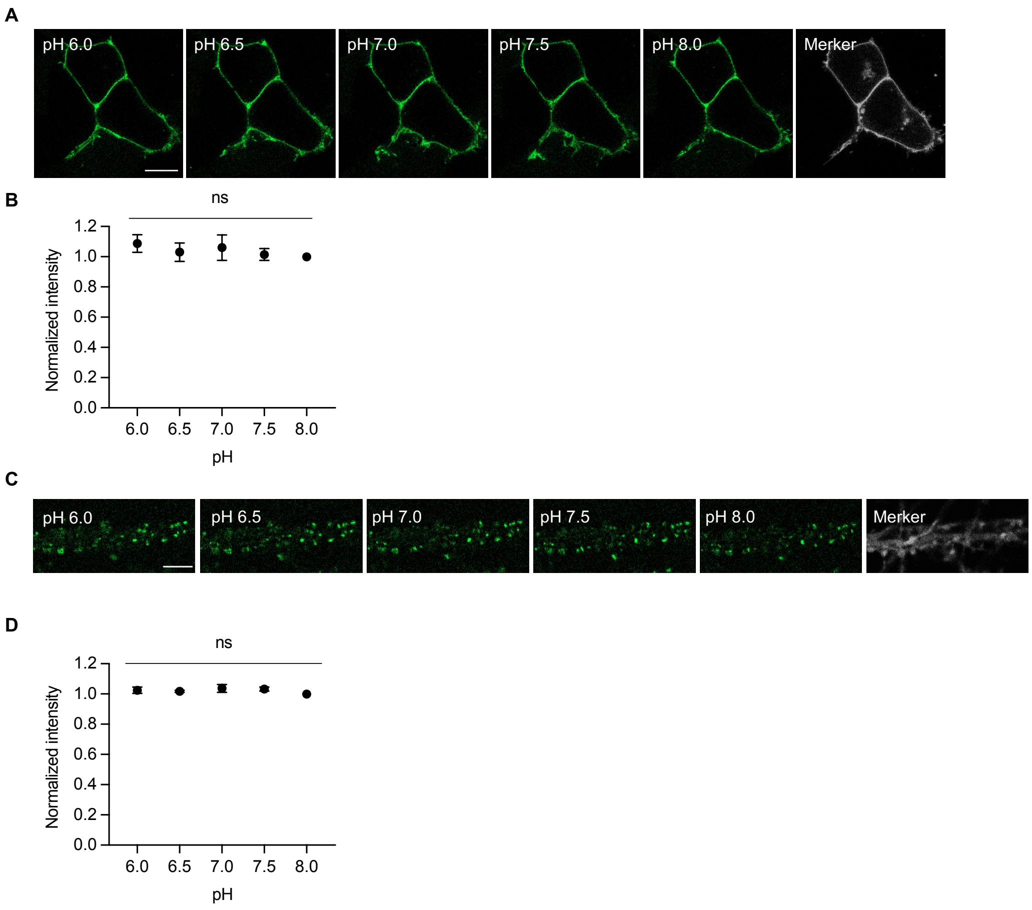

Fig. S13. pH-dependency of PFQX1(AF488) fluorescence on cell surface. (A, C) Representative confocal live images of HEK293T cells expressing GluA2 in **A** and cultured neurons in **C**. pH of the extracellular solution was altered from 6.0 to 8.0. [PFQX1(AF488)] = 100 nM. Scale bar, 10 μm. **(B, D)** The surface intensity was quantified and normalized to the intensity at pH8.0 (n = 3). ns, not significant ($p > 0.05$, One-way ANOVA with Tukey's test). Data are represented as mean ± s.e.m.

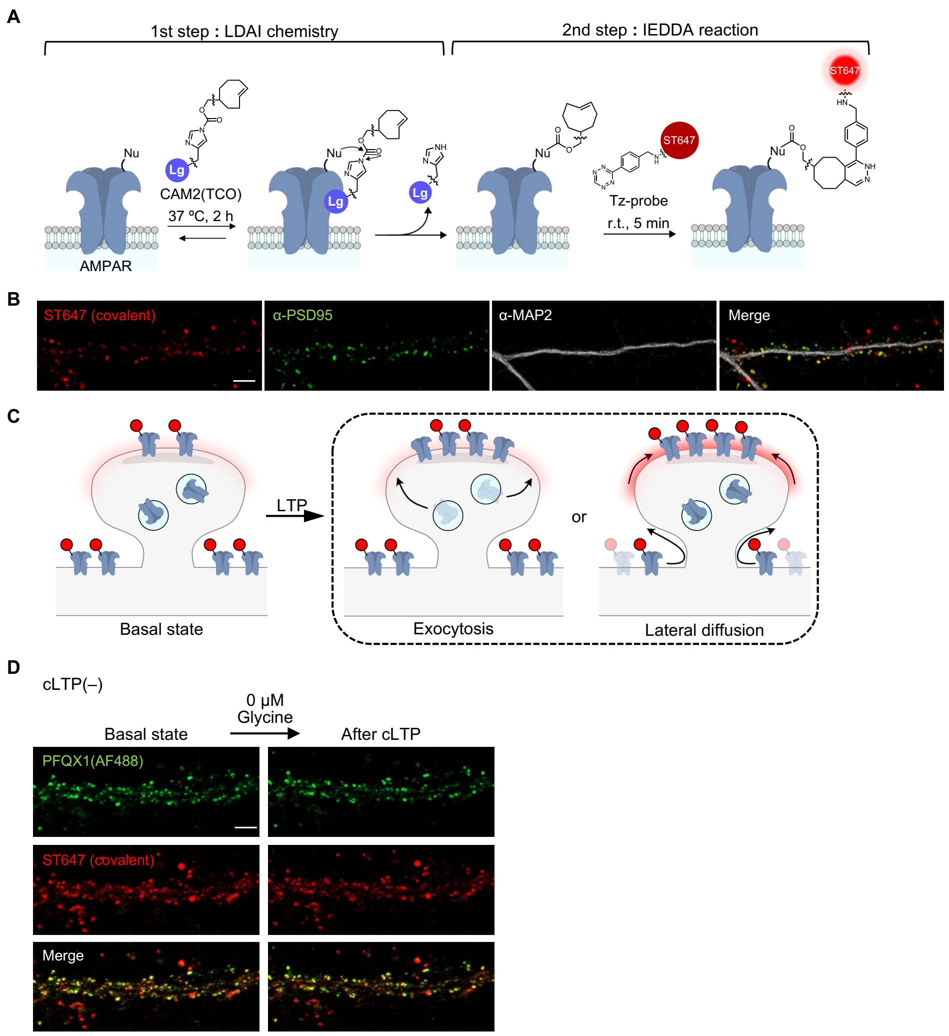

Fig. S14. Covalent ST647 labeling by ligand-directed two-step labeling and evaluation of fluorescent changes after cLTP stimulation. (A) Schematic illustration of ligand-directed two-step labeling method. In the first step, a strained alkene (TCO) is covalently attached to AMPARs. In the second step, Tz(ST647) is selectively tethered to TCO via IEDDA reaction. **(B)** Immunostaining of cultured hippocampal neurons after labeling with ST647. The cells were immunostained with anti-PSD95 (green signal) and anti-MAP2 (gray signal). [CAM2(TCO)] = 2 μM, [Tz(ST647)] = 100 nM. Scale bar, 5 μm. **(C)** A schematic illustration of putative results of AMPAR trafficking after cLTP stimulation, where cell-surface AMPARs are covalently labeled with ST647 by two-step labeling. If exocytosis is the main mechanism of synaptic accumulation, non-labeled AMPARs would be exocytosed, resulting in no change in the intensity of ST647 in spines. If lateral diffusion is the main mechanism, labeled AMPARs would be accumulated, resulting in an enhanced intensity of ST647 in spines. **(D)** Representative live cell images of cLTP experiment under control conditions. Glycine was not added to the cells. The

experimental procedure and analyzed data are shown in Fig. 6C and E, respectively. Scale bar, 5 μm.

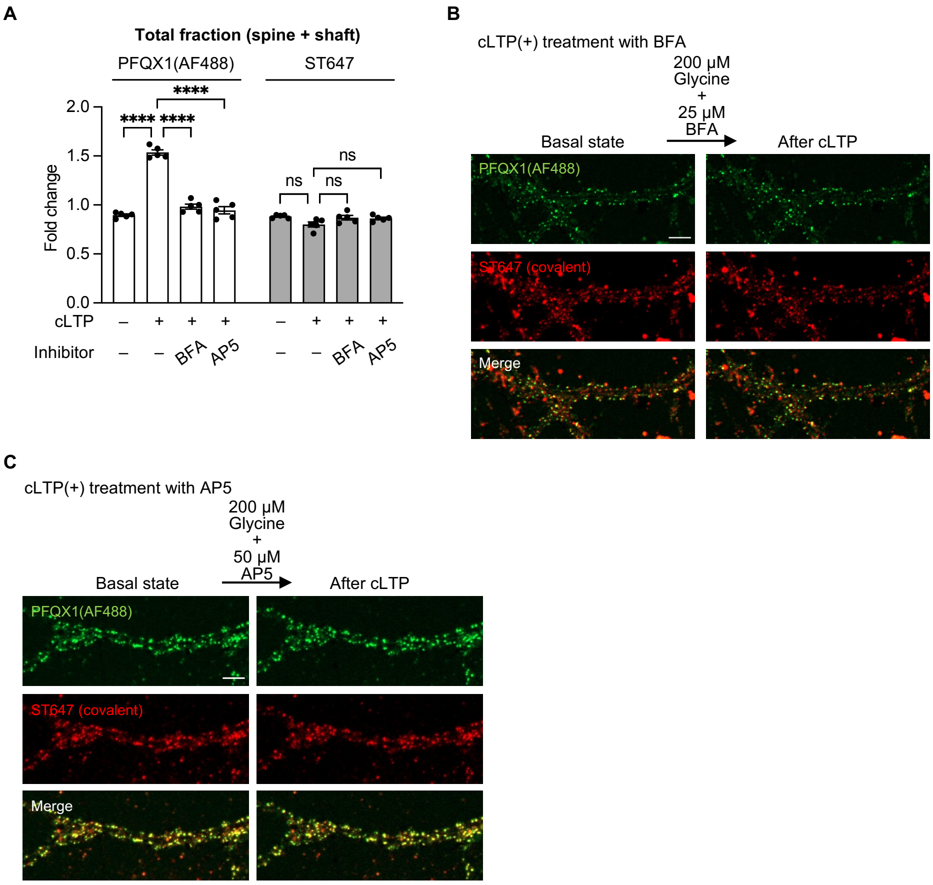

Fig. S15. Increase in cell surface AMPARs in response to cLTP is suppressed by the treatment of inhibitors. (A) The intensity of AF488 and ST647 of the whole cell surface was quantified, with the intensity of "after cLTP" images divided by that of "basal state" images (n = 5). ROIs were placed on the cell surface containing both dendritic spines and dendritic shafts. **(B, C)** Representative live cell images of cLTP experiment treated with inhibitors. [AP5] = 50 μM, [Brefeldin A (BFA)] = 25 μM. Scale bars, 5 μm. ****Significant difference ($p < 0.0001$), ns, not significant ($p > 0.05$, Two-way ANOVA with Tukey's test.). Data are represented as mean ± s.e.m.

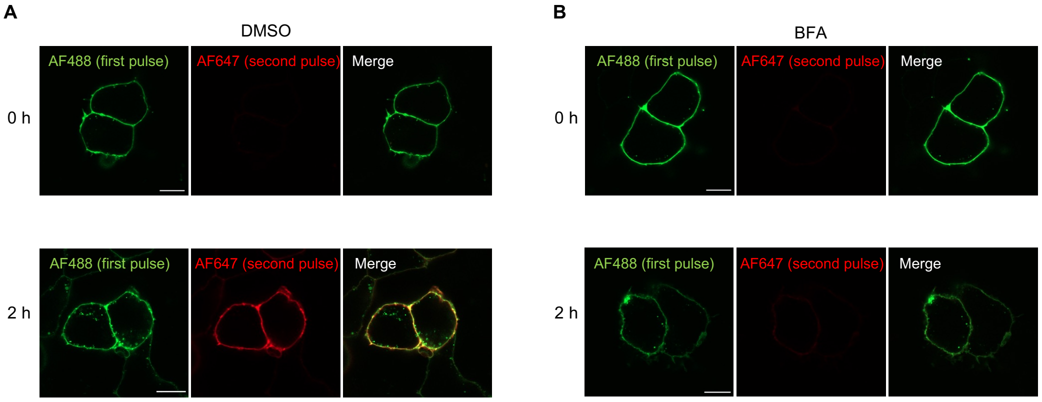

Fig. S16. Inhibition of spontaneous exocytosis by brefeldin A in HEK293T cells. (A, B) Confocal images of HEK293T cells expressing Halo-tag fused GluA2. The cells were treated with 500 nM HTL(AF488) as a first pulse to label cell surface receptors. After incubation with DMSO in **A** or brefeldin A (BFA) in **B** for 2 h at 37 ºC, 500 nM HTL(AF647) was treated as a second pulse to label spontaneously exocytosed receptors. The cells were fixed with 4% PFA. [BFA] = 25 µM. Scale bars, 10 µm.

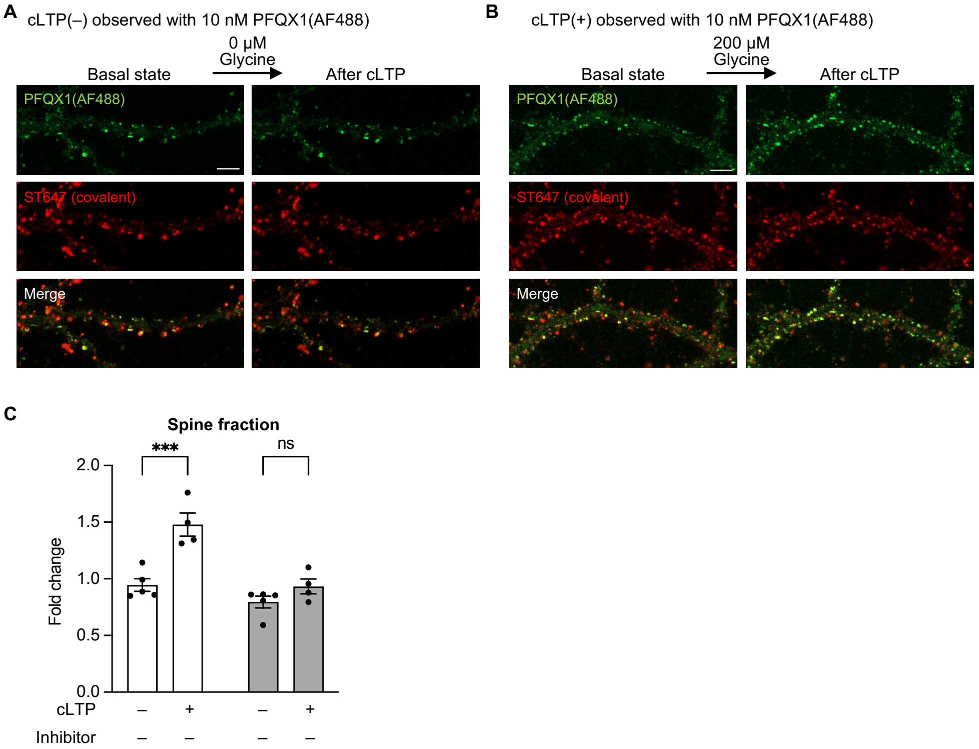

Fig. S17. The short-term inhibition of AMPARs by 10 nM PFQX1(AF488) does not affect AMPAR trafficking. (A, B) Representative live cell images of cLTP experiment visualized with 10 nM PFQX1(AF488). Scale bars, 5 μm. **(A)** Control condition in which glycine was not treated. **(B)** The cells were treated with 200 μM glycine to induce cLTP. **(C)** The intensity of AF488 and ST647 in spines was quantified, with the intensity of "after cLTP" images divided by that of "basal state" images (n = 5). ***Significant difference ($p < 0.001$), ns, not significant ($p > 0.05$, Two-way ANOVA with Sidak's test.). Data are represented as mean ± s.e.m.

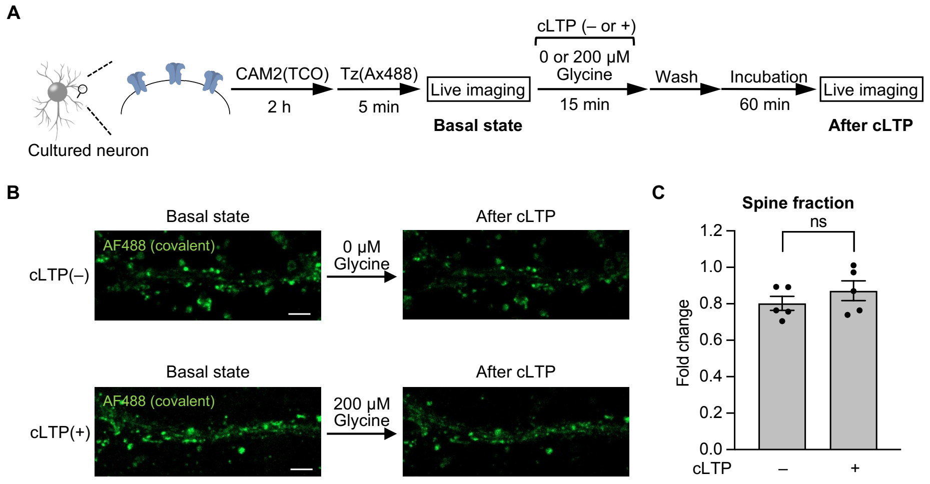

Fig. S18. The fluorescent dye had no effect on the trafficking of AMPARs. (A) Schematic illustration of the experimental procedure. Cultured hippocampal neurons were labeled with 2 μM CAM2(TCO) for 2 h followed by Tz(Ax488) for 5 min. The cells were scanned before cLTP as "basal state" images. The cells were treated with 200 μM glycine for 15 min, and further incubated for 60 min. Then, the cells were scanned as "after cLTP" images. **(B)** Representative images of confocal live cell imaging covalently modified with AF488 are shown. Upper, images of the control condition where glycine was not added to the cells are shown. Lower, images of the cells stimulated with glycine are shown. Scale bars, 5 μm. **(C)** The intensity of AF488 in spines was analyzed, with the intensity of "after cLTP" images divided by that of "basal state" images ($n = 5$). ns, not significant ($p > 0.05$, Student's t-test.). Data are represented as mean ± s.e.m.

Table S1. Properties of PFQX(Dye)

Solubility and affinity to GluA2

	Affinity (nM)[a]	Solubility (μM)[b]
PFQX1(Fl)	4.3 ± 0.5	19.1 ± 0.5
PFQX1(TAMRA)	n.d.	82.6 ± 2.9
PFQX1(AF488)	37.2 ± 9.8	> 1,470 ± 166
PFQX2(Fl)	346 ± 99	472.8 ± 18.2

[a]Affinity was evaluated in HEK293T cells expressing GluA2^{flip}(Q). Detailed results are shown in Fig. 1E and fig. S3A. [b]PFQX(Dye)s were dissolved in HBS.

Affinity of PFQX1(AF488) to AMPARs.

	Affinity (nM)
GluA1^{flip}(Q)	78.7 ± 15.4
GluA2^{flip}(Q)	37.2 ± 9.8
GluA3^{flip}(Q)	30.6 ± 7.0
GluA4^{flip}(Q)	20.2 ± 1.2
GluA2^{flip}(R)	32.3 ± 4.7
GluA2^{flip}(R) + GSG1L	34.5 ± 12.4
GluA2^{flip}(R) + TARP γ7	118.3 ± 44.7
GluK2(Q)	n.d.
Native AMPARs[c]	97.5 ± 9.9

[c]Affinity to native AMPARs were evaluated using primary cultured hippocampal neurons.

Table S2. Data collection and refinement statistics

	S1S2J-PFQX1(AF488)
**Data collection**	
Wavelength	1.000
Resolution range (Å)	47.33 – 1.82 (1.89 – 1.82)
Space group	$P6_122$
Unit cell (a, b, c, α, β, γ) (Å, º)	69.9, 69.9, 227.5, 90, 90, 120
Unique reflections	30612 (2985)
Multiplicity	71.2 (44.4)
Completeness (%)	100.0 (100.0)
Mean $I/\sigma I$	40.92 (5.05)
$R_{merge}{}^{a}$	0.181 (3.369)
$CC_{1/2}$	1.000 (0.983)
**Refinement**	
Reflections for refinement	30604
$R_{work}{}^{b}/R_{free}{}^{c}$	0.169 / 0.200
RMSDs	
lengths (Å)	0.010
angles (º)	1.078
Ramachandran (%)	
favored	99.22
allowed	0.78
outliers	0
Rotamer outliers (%)	0
C–beta outliers (%)	0
Number of non-hydrogen atoms	
S1S2J	2040
waters	237
PFQX1(AF488)	62
sulfates	10
polyethylene glycols	14
Mean B value (Å^2)	
overall	33.92
S1S2J	32.61
waters	40.50
PFQX1(AF488)	39.79
sulfates	71.58
polyethylene glycols	60.24

[a] $R_{merge} = \Sigma_{hkl}\Sigma_i|I_i(hkl) - \langle I_i(hkl)\rangle|/\Sigma_{hkl}\Sigma_i I_i(hkl)$, where i is the number of observations of a given reflection and I(hkl) is the average intensity of the i observations. [b] $R_{work} = \Sigma_{hkl}||F_{obs}(hkl)| - |F_{calc}(hkl)||/\Sigma_{hkl}|F_{obs}(hkl)|$. [c] R_{free} was calculated with a 5% fraction of randomly selected reflections evaluated from refinement. The highest resolution shell is shown in parentheses.

Table S3. Interactions between PFQX1(AF488) and S1S2J

residues	atom type	ligand	atoms[a]	distance (Å)	interaction
E20 (E402)	Cγ	PFQX	F1	3.1	multipolar F amide
Y68 (Y450)	phenyl	PFQX	quinoxaline	3.6	π-stacking
Y68 (Y450)	phenyl	PFQX	quinoxaline	4.1	π-stacking
P96 (P478)	O (main chain)	PFQX	N4	2.8	H-bond
T98 (T480)	N (main chain)	PFQX	O2	2.8	H-bond
R103 (R485)	Nη1	PFQX	O2	2.7	H-bond
R103 (R485)	Nη2	PFQX	O3	2.9	H-bond
S149 (S654)	N (main chain)	PFQX	O5	2.9	H-bond
S149 (S654)	Oγ	PFQX	O4	2.7	H-bond
Y227 (Y732)	OH	PFQX	F2	3.1	H-bond
water122	O	PFQX	O5	3.1	H-bond
water186	O	PFQX	O4	2.6	H-bond
water69	O	PFQX	N2	3.2	H-bond
T181 (T686)	N (main chain)	AF488	O1	2.8	H-bond
T181 (T686)	Oγ	AF488	O1	2.7	H-bond
L145 (L650)	Cδ	AF488	C8	3.9	hydrophobic
R179 (R684)	Nη1	AF488	O8	2.7	salt bridge
T180 (T685)	methyl	AF488	phenyl	4.2	CH-π interaction
water113	O	AF488	O6	2.7	H-bond
symmetry mate					
K151 (K656)	Nζ	AF488	O8	3.0	salt bridge
K151 (K656)	Nζ	AF488	O9	3.4	salt bridge
K151 (K656)	Nζ	AF488	O12	3.3	salt bridge
K151 (K656)	Nζ	AF488	O13	3.0	salt bridge
R155 (R660)	Nη2	AF488	O12	3.2	salt bridge
R155 (R660)	Nη2	AF488	O14	3.0	salt bridge
R170 (R675)	Nη1	AF488	O9	3.2	salt bridge
R170 (R675)	Nη1	AF488	O12	3.4	salt bridge
R170 (R675)	O (main chain)	AF488	N6	3.0	H-bond
R170 (R675)	guanidino	AF488	xanthene	3.6	cation-π interaction

[a]Atomic number of PFQX1(AF488) is described in the below structure.

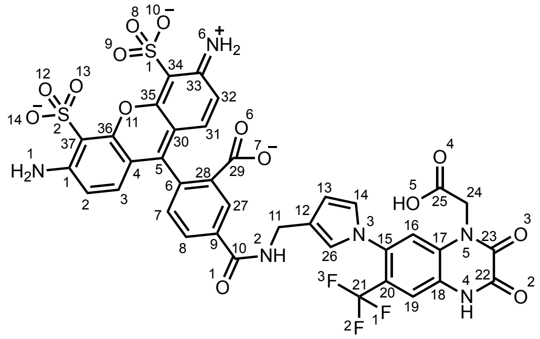

Supplement: Supplementary file 1 — Supplementary Methods Figs. S1 to S18 Tables S1 to S3 [file sciadv.adt6683_sm.pdf]
